# Ontogeny Related Changes in the Pediatric Liver Metabolome

**DOI:** 10.3389/fped.2020.00549

**Published:** 2020-09-29

**Authors:** Christopher M. Wilson, Qian Li, Roger Gaedigk, Charlie Bi, Saskia N. de Wildt, J. Steven Leeder, Brooke L. Fridley

**Affiliations:** ^1^Department of Biostatistics and Bioinformatics, Moffitt Cancer Center, Tampa, FL, United States; ^2^Health Informatics Institute, University of South Florida, Tampa, FL, United States; ^3^Division of Clinical Pharmacology, Toxicology and Therapeutic Innovation, Children's Mercy Hospital, Kansas City, MO, United States; ^4^Department of Pharmacology and Toxicology, Radboud University Medical Center, Nijmegen, Netherlands; ^5^Intensive Care and Department of Pediatric Surgery, Erasmus Medical Center Sophia Children's Hospital, Rotterdam, Netherlands

**Keywords:** childhood development, metabolites, ontogeny, liver, bioinformatics

## Abstract

**Background:** A major challenge in implementing personalized medicine in pediatrics is identifying appropriate drug dosages for children. The majority of drug dosing studies have been based on adult populations, often with modification of the dosing for children based on size and weight. However, the growth and development experienced by children between birth and adulthood represents a dynamically changing biological system, with implications for effective drug dosing, efficacy as well as potential drug toxicity. The purpose of this study was to apply a metabolomics approach to gain preliminary insights into the ontogeny of liver function from newborn to adolescent.

**Methods:** Metabolites were measured in 98 post-mortem pediatric liver samples in two experiments 3 batches of samples, allowing for both technical and biological validation. After extensive quality control, imputation and normalization, non-parametric tests were used to determine which metabolite levels differ between the four age groups (AG) ranging in age from newborn to adolescent (AG1—children <1 year; AG2—children with age between 1 and 6 years; AG3—children with age between 6 and 12 years; AG4—children with age between 12 and 18 years). To identify which metabolites had different concentration levels among the age groups, Kruskal-Wallis and Spearman correlation tests were conducted. Pathway analysis utilized the Gamma Method. Correction for multiple testing was completed using Bonferroni correction.

**Results:** We found 41 metabolites (out of 884) that were biologically validated, and of those 25 were technically replicated, of which 24 were known metabolites. For the majority of these 24 metabolites, concentration levels were significantly lower in newborns than in the other age groups, many of which were long chain fatty acids or involved in pyrimidine or purine metabolism. Additionally, we found two KEGG pathways enriched for association with age: betaine metabolism and alpha linolenic acid and linoleic acid metabolism.

**Conclusions:** Understanding the role that ontogeny of childhood liver plays may aid in determining better drug dosing algorithms for children.

## Introduction

Most drugs prescribed for children have not been studied in the relevant pediatric patient population to determine the appropriate dosing regimen, with ~20% of drugs approved by the FDA being labeled for use in children as of 2016 (from FDA website https://www.fda.gov/drugs/resourcesforyou/consumers/ucm143565.htm). Hence, the majority of drug dosing regimens for use in pediatric populations are based on dosing guidelines developed for adults and modified for use in children based on body weight and size of the child. However, children differ from adults in other ways beyond just size or weight, including body composition and organ development (1, 2). The disposition of many drugs is dependent on hepatic factors, such as blood flow and activity of drug-metabolizing enzymes and transporters.

In particular, the activity of many cytochrome P450 (CYP) isoforms, such as CYP3A4, CYP1A2, and CYP2D6, has been shown to be lower in newborns compared to adults, with each having a distinct developmental trajectory (3, 4). Similar findings have been reported for glucuronosyl transferases (5) and the expression of liver transporters (6). In addition to the critical need to understand the ontogeny of drug disposition to aid in age-appropriate dose selection, there needs to be recognition that a child is a dynamically changing biological system. For example, age-dependent changes in organic acid profiles imply that mitochondrial function may change during growth and development, especially relevant in the context of valproate hepatotoxicity (7). Less studied are the developmental trajectories of hepatic pathways that serve as targets of drug action, such as cholesterol biosynthesis (statins) and glucose homeostasis (metformin).

Technological advances in measuring metabolites and the rapid commercialization of novel instrumentation have sped up the adoption of metabolomics in all aspects of basic, population and clinical biomedical research (8, 9). Metabolites are the substrates, cofactors, and products needed for biological pathways and essential for cellular functions. In addition, many endogenous compounds are also substrates for “drug” metabolizing enzymes and transporters. Therefore, the purpose of this study was to characterize the biochemical changes occurring in liver between birth and 18 years of age and gain initial insight into ages/developmental stages that may be associated with altered drug response or increased susceptibility for age-related drug toxicity not apparent from adult data.

## Materials and Methods

### Liver Samples

Postmortem pediatric human liver tissue samples were obtained through the Brain and Tissue Bank for Developmental Disorders at the University of Maryland (Baltimore, MD), the Liver Tissue Cell Distribution System (LTCDS; University of Pittsburgh and University of Minnesota), and XenoTech LLC (Lenexa, KS). The use of these tissues was classified as non-human subject research by the Children's Mercy Hospital Pediatric Institutional Review Board. A replication set of post-mortem liver tissue samples from autopsies of fetuses (from therapeutic abortions or stillbirths) and infants was provided by the Erasmus Medical Center Tissue Bank, Sophia Children's Hospital, Rotterdam, the Netherlands. Tissue was procured at the time of autopsy within 24 h after death, snap-frozen in liquid nitrogen and stored at −80 °C for later research use. The Erasmus Medical Center Research Ethics Board waived the need for formal ethics approval according to the Dutch Law on Medical Research in Humans. Tissue was collected when parental written informed consent for both autopsy and the explicit use of the tissue for research was present. Samples were selected based on the absence of a clinical diagnosis or medications affecting the liver (CMH and Erasmus Medical Center), and tissue that was histologically normal (Erasmus Medical Center). Samples were stratified into four age groups: <1 year of age (age group 1), 1 to <6 years (age group 2), 6 to <12 years (age group 3), and 12–18 years of age (age group 4). In total 98 liver samples were available for metabolomic analysis. Characteristics of the study group are presented in Table 1.

**Table 1:** Summary of pediatric liver samples included in the study.

**Sample set**	**Tissue source**	**Age group**			
		**Age <1 year (AG1)**	**1≤ age <6 years (AG2)**	**6≤ age <12 years (AG3)**	**12≤ age <18 years (AG4)**
All samples	Erasmus Medical Center	20	1	0	2
	Minnesota	0	8	8	12
	Pittsburgh	0	3	10	6
	UMB	13	2	1	1
	XenoTech	1	7	2	1
	Total	34	21	21	22
Samples in Experiment 1, Batch 1 and Experiment 2, Batch 2	Erasmus Medical Center	0	0	0	0
	Minnesota	0	8	6	8
	Pittsburgh	0	0	2	2
	UMB	12	2	1	1
	XenoTech	0	4	1	0
	Total	13	14	10	11
Samples in Experiment 2, Batch 3	Erasmus Medical Center	20	1	0	2
	Minnesota	0	0	2	4
	Pittsburgh	0	3	8	4
	UMB	1	0	0	0
	XenoTech	0	3	1	1
	Total	21	4	11	11

### Metabolomic Analysis

**Figure 1:**
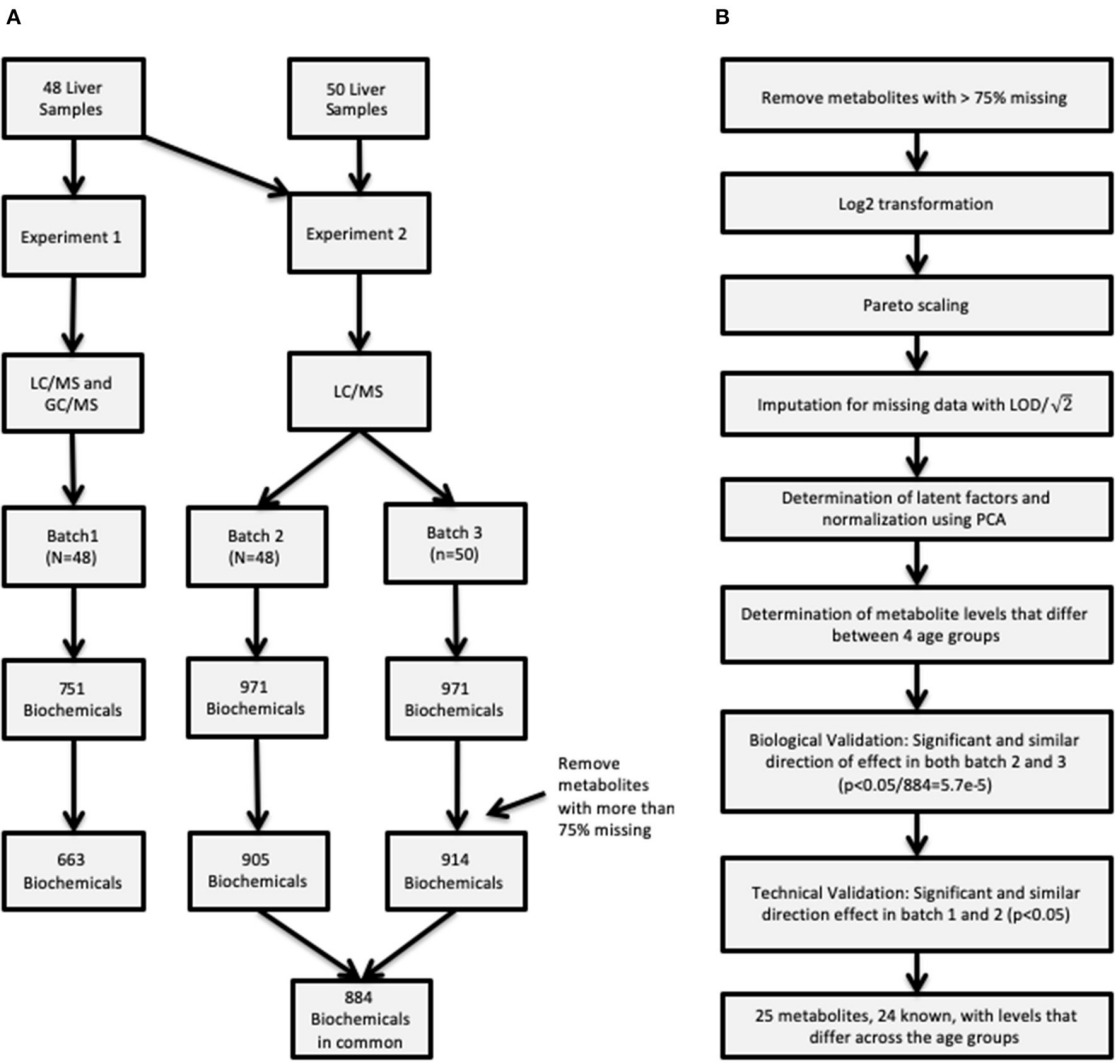
**(A)** Description of the two procedures used to obtain metabolite concentrations and how the samples were allocated in the three batches. **(B)** Flowchart of the entire analysis batch including how the quality control was conducted for the raw data, and summarizes the metabolite validation.

Untargeted metabolomic profiling was conducted by Metabolon Inc. (Durham, NC). Samples in two sets of experiments, Experiment 1 and Experiment 2, as described below and depicted in Figure 1A.

#### Experiment 1

The first experiment was completed using the first set of samples (*N* = 48) (referred to as “batch 1”).

Metabolite extraction and detection as previously described (10). Briefly, liver sample preparation was conducted using a proprietary series of organic and aqueous extractions to remove the protein fraction and optimize recovery of small molecules through the automated MicroLab STAR® system (Hamilton Company, UT, USA), centrifuged, and the resulting supernatants were analyzed by analyzed by ultra-performance liquid chromatography mass spectrometry (UPLC-MS/MS) in a positive and negative ion mode (UPLC: Waters, Milford, MA; mass spectrometer: Thermo-Finnigan LTQ, Thermo Fisher Scientific, Waltham, MA, scan range, 80–1,000 m/z) and by GC-MS (Thermo-Finnigan Trace DSQ fast-scanning single-quadrupole mass spectrometer, scan range 50–750 m/z). The final experiment 1 metabolomic dataset comprised a total of 751 biochemicals, 478 compounds of known identity (named biochemicals) and 273 compounds of unknown structural identity. As initial statistical analysis revealed an age-dependent effect that could not be distinguished from a tissue source-related effect, a replication set of group 1 samples was obtained through collaboration with the Erasmus Medical Center/Sophia Children's Hospital.

#### Experiment 2

Given that the metabolomic platform changed between the first analysis and the sample set containing the replication samples, the second experiment examined the entire set of 98 samples. The same 48 samples previously processed in Experiment 1 and designated as “batch 1” above were re-analyzed on the new platform, with the results designated “batch 2.” The replication samples from the Erasmus Medical Center/Sophia Children's Hospital and additional samples from CMH (*N* = 50) are designated as “batch 3.” Following the sample extraction, the resulting extract was analyzed using a Waters ACQUITY UPLC and a Thermo Scientific Q-Exactive high resolution/accurate mass spectrometer interfaced with a heated electrospray ionization (HESI-II) source and Orbitrap mass analyzer operated at 35,000 mass resolution (11). Four methods were utilized: two separate reverse phase (RP)/UPLC-MS/MS methods with positive ion mode electrospray ionization (ESI), RP/UPLC-MS/MS with negative ion mode ESI, and HILIC/UPLC-MS/MS with negative ion mode ESI. The MS analysis alternated between MS and data-dependent MS^n^ scans using dynamic exclusion. The scan range varied slightly between methods but covered 70–1,000 m/z. The final experiment 2 metabolomic dataset comprised a total of 971 biochemicals, 779 compounds of known identity (named biochemicals) and 192 compounds of unknown structural identity.

#### Data Processing

Raw data was extracted, peak-identified and QC processed using proprietary hardware and software. Compounds were identified by comparison to library entries of purified standards or recurrent unknown entities (12). Peaks were quantified using area-under-the-curve. As the metabolomic assays span multiple days, a data normalization step was performed to correct variation resulting from instrument inter-day tuning differences. Essentially, each compound was corrected in run-day blocks by registering the medians to equal one (1.00) and normalizing each data point proportionately.

#### Statistical Analyses

Additional quality control measures were taken and are outlined in Figure 1B, such as imputation of missing data, and normalization. First, metabolites with more than 75% missing data were removed from subsequent analysis. After removing metabolites from analysis data set, data were log_2_ transformed and scaled followed by imputation of missing values with the minimum detected level for the specific metabolite divided by $\sqrt{2}$. Latent effects are unmeasurable and unobservable factors can bias results, some examples of latent effects are batch effects, day-to-day variations in instrument performance. Latent factors were identified using principal component analysis (PCA), where the top 2 principal components (PCs) were adjusted for by using a linear model for each batch. Removing the top 2 PCs strikes a balance between removing latent technical factors, while the biologically relevant factors are still present. On the other hand it is possible that some of the estimated latent factors are not technical artifacts but rather represent true biology presented in the data.

It is unreasonable to assume that the abundance of each metabolite is normally distributed, hence the rank-based non-parametric Kruskal-Wallis (KW) test was performed, for each batch and metabolite individually, to determine if there were any differences in metabolite levels among the four age groups (AG1—children <1 year; AG2—children with age between 1 and 6 years; AG3—children with age between 6 and 12 years; AG4—children with age between 12 and 18 years). Linear associations between age group and metabolite levels were investigated using Spearman correlation test. To adjust for multiple testing a Bonferroni correction was applied. Statistical analysis was restricted to metabolites in common between the three batches of samples to enable assessment of both technical and biological validation. Biological validation was achieved by determining which metabolites had a significant *p*-value from KW test in both batches 2 and 3. This set of metabolites was then compared to the set of metabolites with a significant *p*-value from KW test (*p* < 0.05) in Batch 1 for technical validation. Pairwise differences were assessed for technically validated metabolite using Mann-Whitney tests.

The implication of an association between the concentrations of a single metabolite and age can be difficult to relate to important biological processes, while grouping metabolites into biologically meaningful sets can help understand an entire metabolite set. To determine association between age and a metabolite set we used the Gamma Method, a variation of Fisher's method (13). In the Gamma method, *p*-values from the metabolite-age association analysis were combined into a test statistic defined as $\sum_{i=1}^{k}h\left(p_{i}\right)$, where *k* represents the number of metabolites in a pathway, *p*_*i*_ represents the *p*-value for the association between the *i*th metabolite and age group, and *h*() represent the inverse gamma cumulative distribution function [i.e., ${G_{\omega ,1\;}}^{-1}\left(1-p_{i}\right)$]. This transformation gives more weight with *p*-values below a soft truncation threshold value determined by the shape parameter ω (13, 14). For this analysis we set the shape parameter to 0.0382, which gives more weight to *p* < 0.1. Due to the correlation between metabolites, the sum of transformed *p*-values does not necessarily follows a known distribution; therefore empirical *p*-values were computing using permutation methods, with *p*-values estimated from 100,000 permutations. Definition and mapping of metabolites to the Kyoto Encyclopedia of Genes and Genomes (KEGG) pathways and drug metabolite sets was completed using pathway mapping data from MetaboAnalyst (15). Metabolite pathways with at least 5 metabolites and coverage of at least 50% were included in the analysis, excluding pathways related to disease processes, resulting in 2 KEGG and 30 drug metabolite pathways being analyzed. Only metabolites that were present all three batches were considered for pathway analysis. Pathways were considered enriched if the empirical *p*-value derived using the Gamma method is <0.01 in all three batches.

## Results

**Figure 2:**
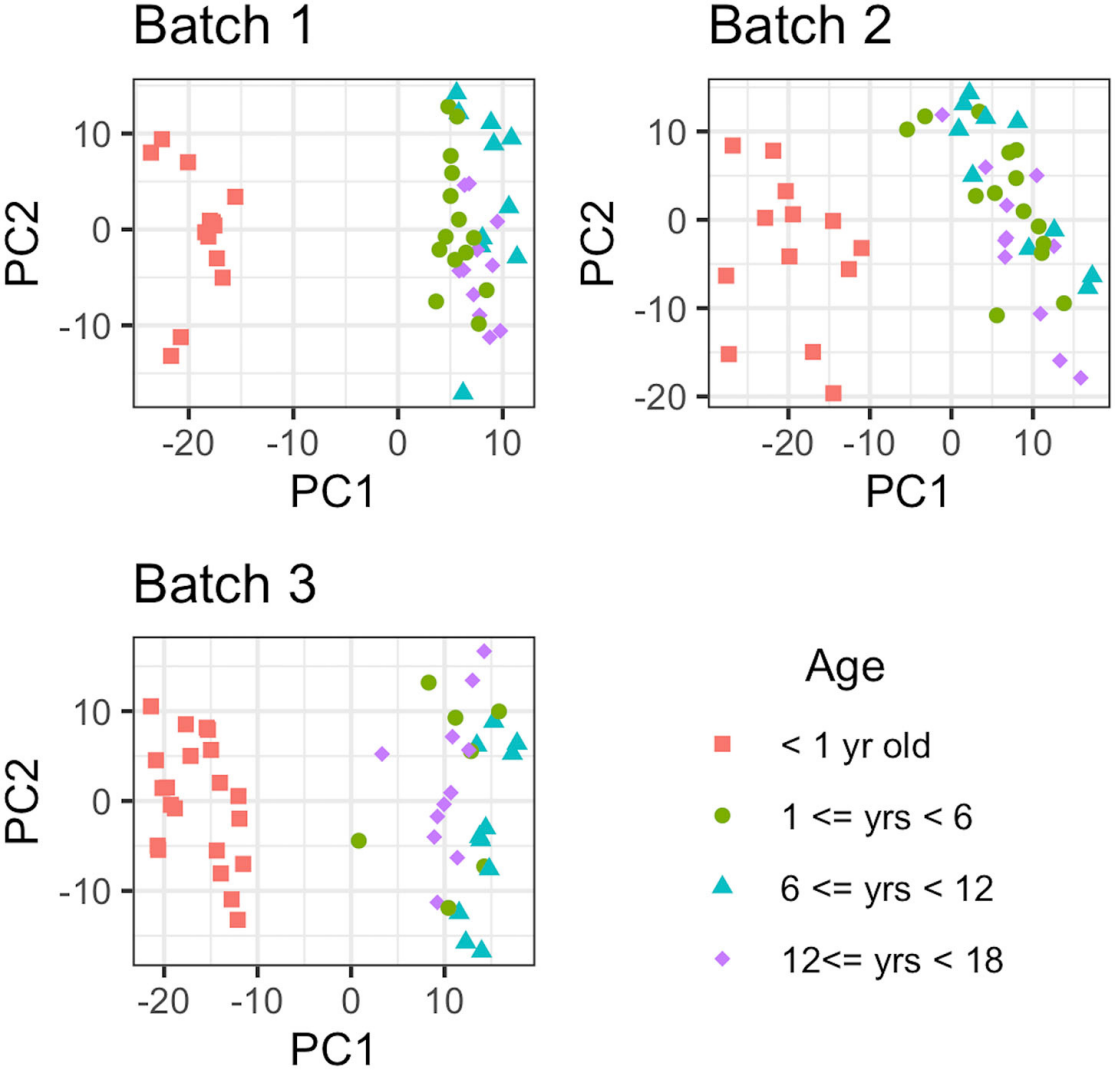
Principal Component Analysis of each metabolite for each batch. The metabolomic liver profiles for children <1 year old clearly separate from the profiles for the children older than 1 year of age in PCA plot.

Metabolites with more than 75% missing data (across all samples) were removed from the analyses; 88 out of 751 metabolites were removed from consideration from batch 1, while 66 and 57 out of 971 metabolites were not considered in batches 2 and 3, respectively. After the removal of metabolites with >75% missing values, there were 322 metabolites in common between the 3 batches for comparative analysis (884 in common between batch 2 and batch 3). First, the effect due to age was removed from the data and then PCA was conducted to estimate latent features. Then, the first 2 principal component removed for each batch individually, with the resulting data presented in Figure 2. As Figure 2 illustrates, there is a large difference in global metabolites levels between the infant (AG1) and the early childhood/late childhood/adolescent age groups (AG2–AG4). The 2 principal components that were removed consisted of 29.83, 26.34, and 27.33% of the total variance in each batch, respectively. There were 16, 21, and 11 metabolites that were present in only AG1 in batch 1, 2, and 3, respectively. However, there was only one metabolite, estriol 3-sulfate, that was only present in AG1 for all batches.

Assessment of the association of each of the 884 metabolites with age group in Batch 2 and Batch 3 utilizing the KW test detected 70 and 197 metabolites in batch 2 and batch 3, respectively (Bonferroni adjusted *p* < 0.05/884 = 5.7e-5) (Supplemental Tables 1, 2). Spearman correlation tests to determine linear trends between metabolites and the four age groups detected 95 and 286 metabolites in batch 2 and 3, respectively (*p* < 0.05/884). Under the null hypothesis that a metabolite is not associated with age and independent between tests (i.e., metabolites), we would have expected to have 0 metabolites detected at the 5.7e-5 significance level. Thus, there appears to be a significant departure from the null hypothesis. Forty-one of these metabolites were detected in both batch 2 and batch 3 and had similar direction of effect as measured by Spearman correlation (Rho), were thus considered biologically validated.

**Figure 3:**
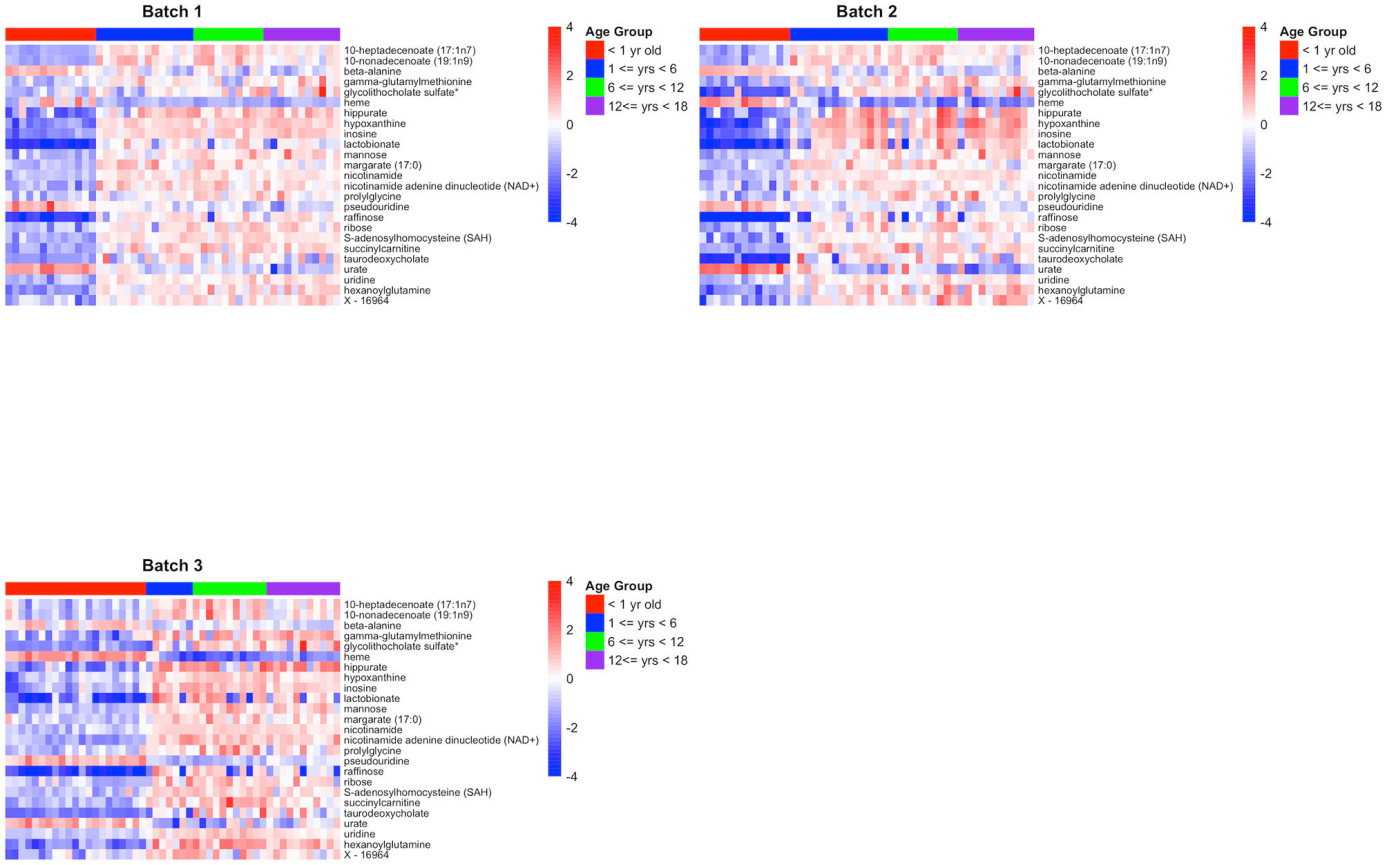
Heatmaps for the 25 biologically and technically validated metabolites for each of the three batches.

For technical validation, 25 of these 41 biologically-validated metabolites were also associated with age in batch 1 (*p* < 0.05), of which 24 are known metabolites (Supplemental Table 3). The 24 known metabolites are presented in Table 2, with a heatmap of the 25 metabolite levels presented in Figure 3 and Supplemental Figure 1. Of the 25 metabolites, 20 were found to have increasing levels over childhood development with the lowest levels present in the infant age group (AG1) and little difference in metabolite levels between the three older age groups. Additionally, pairwise comparison between the four age groups revealed that in many cases the distribution of metabolites levels varied significantly between infants and all other age groups (*p* < 0.001; Table 2). Of the 24 known metabolites found to be associated with age, 3 metabolites were in the sub-pathways “purine metabolism (hypo)xanthine/Inosine containing,” “pyrimidine metabolism, uracil containing,” and “long fatty acids.”

**Table 2:** Twenty-four known metabolites out of the 25 biologically and technically replicated metabolites.

**Metabolite**	**HMDB**	**Super pathway**	**Sub pathway**	**Batch**	**Median of normalized data**				**Four age group comparison**			**Pairwise comparisons**		
									**KW test**	**Spearman test**		**Mann-Whitney** ***p*** **<** 0.001^*^		
					**Infant (AG1)**	**Early childhood (AG2)**	**Late childhood (AG3)**	**Adolescent (AG4)**	***p*-value**	**Rho**	***p*-value**	**AG1 vs. AG2**	**AG1 vs. AG3**	**AG1 vs. AG4**
10-heptadecenoate (17:1n7)	HMDB60038	Lipid	Long chain fatty acid	1	−1.30	0.46	0.87	0.38	3.13E-07	0.74	1.42E-09	x	x	x
				2	−1.16	0.55	0.60	0.31	2.41E-06	0.65	6.94E-07	x	x	x
				3	−0.75	0.22	0.91	0.01	1.34E-05	0.68	6.58E-08			x
10-non-adecenoate (19:1n9)	HMDB13622	Lipid	Long chain fatty acid	1	−1.20	0.46	0.71	0.01	6.56E-07	0.66	3.15E-07	x	x	x
				2	−1.08	0.78	0.69	0.08	1.16E-06	0.55	4.58E-05	x		x
				3	−0.87	0.62	0.86	0.11	2.08E-05	0.64	4.69E-07			x
Margarate (17:0)	HMDB02259	Lipid	Long chain fatty acid	1	−0.98	0.53	0.31	0.09	1.72E-06	0.62	2.11E-06	x	x	x
				2	−0.84	0.56	0.71	−0.08	3.29E-06	0.52	1.55E-04	x		x
				3	−0.72	0.56	0.70	0.15	1.66E-05	0.66	1.70E-07			x
Taurodeoxycholate	HMDB00951	Lipid	Primary bile acid metabolism	1	−1.55	0.10	−0.10	0.00	1.60E-04	0.51	2.18E-04	x		x
				2	−3.18	0.20	−0.09	0.13	6.21E-06	0.57	2.12E-05	x	x	x
				3	−2.17	−0.04	0.10	0.20	1.61E-06	0.76	1.39E-10		x	x
Beta-alanine	HMDB00056	Nucleotide	Pyrimidine metabolism, uracil containing	1	1.00	−0.53	0.23	−0.58	8.71E-05	−0.48	5.02E-04	x	x	
				2	1.03	−0.36	−0.15	−0.75	2.60E-05	−0.50	2.77E-04	x	x	
				3	0.91	−0.62	−0.18	−0.74	1.63E-04	−0.48	4.63E-04			
Pseudouridine	HMDB00767	Nucleotide	Pyrimidine metabolism, uracil containing	1	0.95	−0.04	−0.43	−0.68	3.90E-06	−0.69	4.25E-08	x	x	x
				2	0.97	−0.38	−0.28	−0.63	4.03E-05	−0.57	2.65E-05	x	x	x
				3	1.09	−0.79	−1.14	−0.36	1.34E-08	−0.82	5.61E-13	x	x	x
Uridine	HMDB00296	Nucleotide	Pyrimidine metabolism, uracil containing	1	−0.97	0.42	0.33	0.45	2.88E-06	0.60	7.99E-06	x	x	x
				2	−0.96	0.24	0.05	0.35	2.73E-05	0.51	2.45E-04	x	x	x
				3	−0.77	0.58	0.82	0.38	1.34E-08	0.85	3.83E-15	x	x	x
Hypoxanthine	HMDB00157	Nucleotide	Purine metabolism, (Hypo)Xanthine/Inosine containing	1	−1.83	0.66	0.73	0.77	1.81E-06	0.64	1.20E-06	x	x	x
				2	−2.71	1.07	1.00	1.40	9.45E-06	0.58	1.51E-05	x	x	x
				3	−0.58	0.21	0.91	0.43	8.54E-08	0.82	2.95E-13	x	x	x
Inosine	HMDB00195	Nucleotide	Purine metabolism, (Hypo)Xanthine/Inosine containing	1	−1.70	0.60	0.73	0.82	2.53E-06	0.65	4.88E-07	x	x	x
				2	−2.17	0.78	0.77	1.37	3.13E-06	0.60	6.54E-06	x	x	x
				3	−1.16	0.83	1.07	0.57	8.44E-08	0.80	4.07E-12	x	x	x
Urate	HMDB00289	Nucleotide	Purine metabolism, (Hypo)Xanthine/Inosine containing	1	1.47	−0.37	−0.54	−1.09	1.31E-06	−0.65	5.23E-07	x	x	x
				2	2.04	−0.83	−0.38	−1.40	6.32E-06	−0.56	2.94E-05	x	x	x
				3	1.03	−1.19	0.65	−0.48	1.43E-05	−0.64	5.04E-07		x	x
S-adenosylhomocysteine (SAH)	HMDB00939	Amino acid	Methionine, cysteine, SAM, and taurine metabolism	1	−1.21	0.39	0.57	0.34	2.82E-06	0.67	1.65E-07	x	x	x
				2	−1.28	0.22	0.07	0.03	2.07E-05	0.54	7.29E-05	x	x	x
				3	−0.91	0.75	0.75	0.43	2.30E-06	0.68	4.18E-08	x	x	x
Gamma-glutamylmethionine	HMDB29155	Peptide	Gamma-glutamyl amino acid	1	−0.69	0.31	−0.13	0.34	4.92E-04	0.33	2.35E-02	x	x	
				2	−1.20	0.21	0.67	0.41	1.88E-05	0.68	8.57E-08	x	x	x
				3	−1.40	1.08	0.88	1.19	3.10E-06	0.66	2.08E-07		x	x
Prolylglycine	HMDB11178	Peptide	Dipeptide	1	−0.78	−0.13	0.55	0.14	5.95E-05	0.68	1.10E-07		x	x
				2	−0.76	−0.19	0.84	0.14	3.17E-05	0.71	1.97E-08			x
				3	−0.93	0.39	0.73	0.38	3.65E-05	0.65	2.53E-07			x
Nicotinamide	HMDB00902	Cofactors and vitamins	Nicotinate and nicotinamide metabolism	1	−0.92	0.44	0.28	0.41	3.34E-06	0.57	2.80E-05	x	x	x
				2	−0.87	0.44	0.21	0.52	1.55E-06	0.51	1.90E-04	x	x	x
				3	−0.74	0.64	0.76	0.54	3.90E-08	0.83	1.52E-13	x	x	x
Nicotinamide adenine dinucleotide (NAD+)	HMDB00229	Cofactors and vitamins	Nicotinate and nicotinamide metabolism	1	−1.50	0.47	0.54	0.25	2.89E-06	0.56	3.64E-05	x	x	x
				2	−0.88	0.43	0.36	0.08	1.97E-05	0.48	5.52E-04	x	x	x
				3	−1.17	0.72	0.95	0.67	7.02E-08	0.78	2.25E-11	x	x	x
Glycolithocholate sulfate^*^	HMDB02639	Cofactors and vitamins	Secondary bile acid metabolism	1	−1.02	−0.05	0.22	0.81	9.35E-03	0.48	5.51E-04			
				2	−2.46	0.16	0.53	0.74	1.55E-05	0.68	9.77E-08	x	x	x
				3	−1.85	−0.10	0.78	0.59	6.76E-07	0.79	1.38E-11		x	x
Heme	HMDB03178	Cofactors and vitamins	Hemoglobin and porphyrin metabolism	1	−0.49	−1.07	−1.31	−1.10	7.81E-04	−0.54	6.87E-05			
				2	1.16	−1.68	−1.95	−1.64	3.22E-06	−0.67	2.25E-07	x	x	x
				3	1.64	−1.82	−2.26	−1.58	1.71E-08	−0.86	1.52E-15	x	x	x
Hippurate	HMDB00714	Xenobiotics	Benzoate metabolism	1	−2.15	0.75	0.66	0.93	4.90E-05	0.59	8.63E-06	x	x	x
				2	−1.99	0.63	0.76	1.25	5.32E-05	0.58	1.54E-05	x	x	x
				3	−1.75	1.47	1.36	1.96	4.45E-06	0.64	7.07E-07	x	x	x
Lactobionate		Carbohydrate	Disaccharides and oligosaccharides	1	−3.12	0.16	−0.03	−0.21	3.04E-06	0.57	1.93E-05	x	x	x
				2	−3.46	0.63	0.45	0.54	1.22E-05	0.54	7.82E-05	x	x	x
				3	−3.08	1.12	1.29	0.23	2.29E-05	0.63	8.42E-07		x	
Mannose	HMDB00169	Carbohydrate	Fructose, mannose, and galactose metabolism	1	−1.25	0.23	0.51	0.64	2.57E-06	0.71	2.18E-08	x	x	x
				2	−0.97	0.17	0.38	0.52	2.52E-06	0.66	3.91E-07	x	x	x
				3	−0.94	0.57	0.92	0.66	1.50E-07	0.80	2.28E-12	x	x	x
Raffinose	HMDB03213	Carbohydrate	Disaccharides and oligosaccharides	1	−2.87	0.47	0.11	0.34	7.21E-06	0.55	5.55E-05	x	x	x
				2	−1.05	0.07	0.62	−0.04	7.20E-06	0.56	3.41E-05	x	x	x
				3	−1.01	0.48	0.68	−0.42	1.01E-05	0.66	1.66E-07		x	x
Ribose	HMDB00283	Carbohydrate	Pentose metabolism	1	−0.92	0.40	0.84	0.41	1.81E-05	0.67	2.40E-07	x	x	x
				2	−1.05	0.26	0.62	0.42	5.04E-05	0.62	2.17E-06	x	x	x
				3	−1.01	0.22	0.7*s*3	0.52	1.03E-05	0.70	1.69E-08		x	x
Hexanoylglutamine				1	−1.60	0.33	0.42	0.83	1.81E-06	0.70	2.68E-08	x	x	x
				2	−1.49	0.54	0.43	0.79	5.18E-06	0.64	9.67E-07	x	x	x
				3	−1.88	0.57	1.54	0.74	3.89E-08	0.84	3.05E-14	x	x	x
Succinylcarnitine	HMDB61717	Energy	TCA cycle	1	−1.31	0.32	0.95	0.61	1.33E-06	0.74	1.38E-09	x	x	x
				2	−1.24	0.40	0.96	0.36	5.27E-06	0.66	3.28E-07	x	x	x
				3	−1.17	0.68	1.22	0.20	3.64E-08	0.83	1.07E-13	x	x	x

* *No Mann-Whitney Tests between Early Childhood, Late Childhood or Adolescent had p < 0.001*.

**Figure 4:**
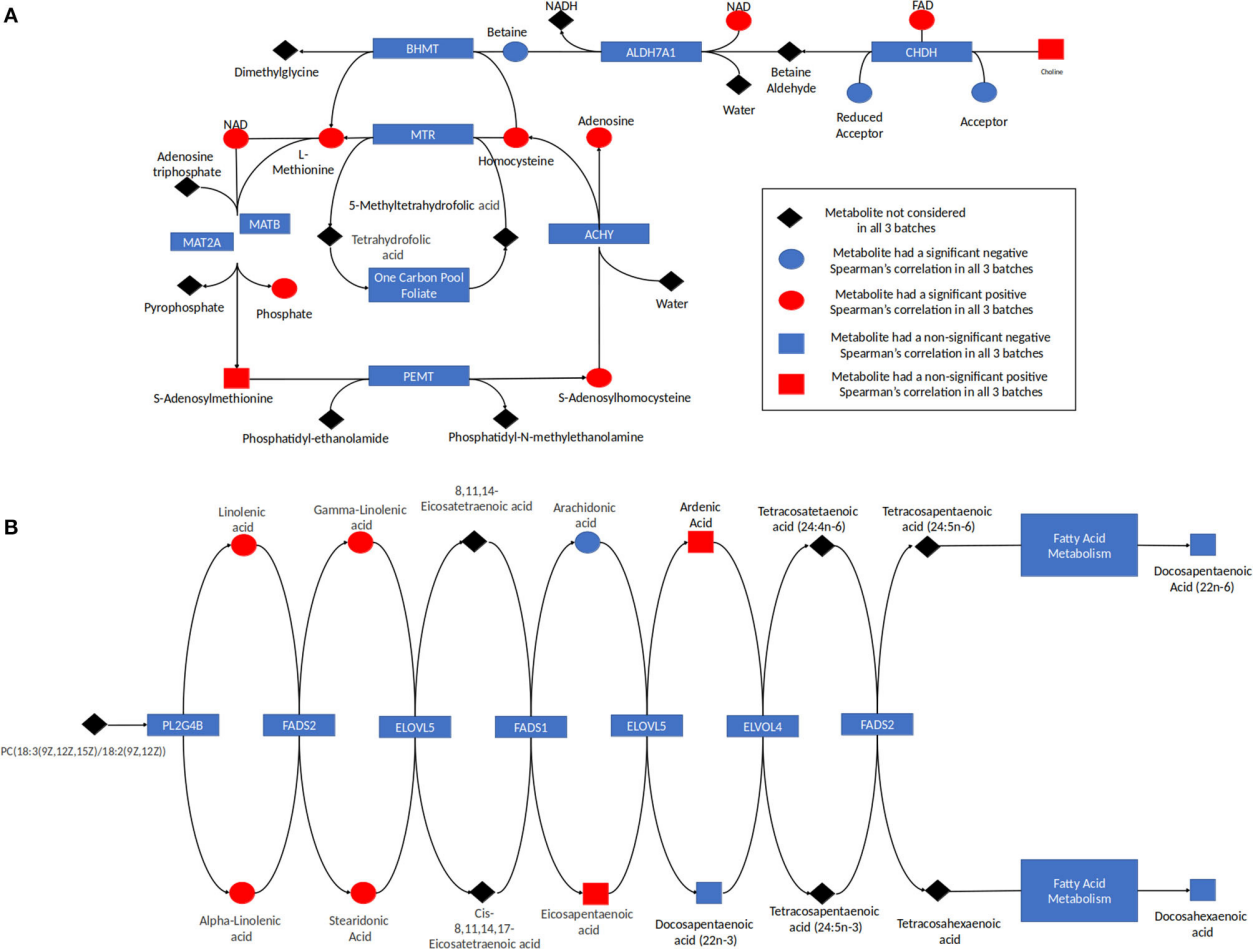
**(A)** Betaine pathway and **(B)** alpha linolenic acid and Linoleic acid metabolism. The results for metabolites measured in this study are indicated with red indicating the metabolite was increasing with age and blue indicating the metabolite was decreasing in abundance with age. Metabolites in the pathway not measured are indicated in black. The shape corresponds to the significance of the Spearman correlation of metabolite's concentration and age, where a square indicates that the *p* < 0.05 and a circle shows that the *p* > 0.05.

Analysis of the pathways/metabolite sets (removing pathways related to disease etiology) with more than 5 metabolites and at least 50% coverage of the pathway resulted in a total of 32 significant metabolite sets (*p* < 0.001 in all 3 batches) (Supplemental Table 4). Based on the use of 100,000 permutations, the smallest observable empirical *p* < 1/100,000 = 1.0 × 10^−5^. The most significant pathway in all three batches was the “Betaine Metabolism” pathway (Table 3). For this pathway, 12 of the 21 metabolites in this pathway were present in our study, resulting in a *p* < 1.0 × 10^−5^ all three batches. The 12 metabolites included in our analysis are: 5-methyltetrahydrofolate (5MeTHF), adenosine, betaine, choline, dimethylglycine, flavin adenine dinucleotide (FAD), homocysteine, methionine, nicotinamide adenine dinucleotide (NAD+), phosphate, S-adenosylhomocysteine (SAH), S-adenosylmethionine (SAM). The majority of these metabolites were individually significant associated with age. To put these results in reference to the biological pathway, we have plotted them with the pathway in Figure 4A and have denoted the metabolites measured in our study and the results for association with age. The other KEGG pathway associated with age was alpha-linolenic Acid and linoleic acid metabolism (Table 3 and Figure 4B), for which we observed 5 metabolites with *p* < 0.05. These 5 metabolites (arachidonate (20:4n6), docosapentaenoate (n3 DPA; 22:5n3), linoleate (18:2n6), linolenate [alpha or gamma; (18:3n3 or 6)]) were all observed to be increasing in abundance age. For 30 of the 32 metabolism and/or drug related pathways (Supplemental Table 4), the same 10 metabolites (adenosine monophosphate, L-alanine, L-methionine, L-leucine, L-histidine, L-proline, L-asparagine, L-valine, L-threonine, L-soleucine) were observed in the pathways resulting in the same pathway level results for batch 1, batch 2 and batch 3 of *p* = 0.00011, 0.00017, and <1.0 × 10^−5^, respectively (Table 3 and Supplemental Figure 2).

**Table 3:** Summary of results for the measured metabolites in the each of the ontogeny related pathways.

**Pathway**	**Metabolite**	**HMDB**	**Batch 1**		**Batch 2**		**Batch 3**	
			***p*-value**	**Rho**	***p*-value**	**Rho**	***p*-value**	**Rho**
Drug action pathway^*^	**Adenosine monophosphate**	HMDB0000045	0.0012	0.4312	4.00E-04	0.3365	<1E-05	0.662
	L-Alanine	HMDB0000161	8.00E-04	0.5868	0.1277	0.3398	0.0023	0.4674
	**L-Asparagine**	HMDB0000168	0.0833	0.3688	5.00E-04	0.5863	<1E-05	0.7089
	L-Histidine	HMDB0000177	0.1322	0.3261	0.0483	−0.0854	0.2539	0.2396
	**L-Isoleucine**	HMDB0000172	1.00E-04	0.6678	<1E-05	0.6705	0.0019	0.5125
	L-Leucine	HMDB0000687	0.0085	0.488	0.104	0.3392	0.46	0.1514
	**L-Methionine**	HMDB0000696	0.0025	0.5037	3.00E-04	0.6065	1.00E-04	0.6249
	L-Proline	HMDB0000162	0.1229	0.2928	0.1703	0.1925	0.6005	0.0265
	L-Threonine	HMDB0000167	0.3312	0.2193	0.3346	0.2387	0.8871	−0.0465
	L-Valine	HMDB0000883	0.0478	0.4046	0.4396	0.1743	0.9175	−0.0569
Alpha linolenic acid and linoleic acid metabolism	Adrenic acid	HMDB0002226	0.4488	0.0166	0.1268	0.0068	0.0508	−0.074
	**Arachidonic acid**	HMDB0001043	0.0067	−0.2795	0.069	−0.1788	1.00E-04	−0.6536
	8,11,14-Eicosatrienoic acid	HMDB0002925	0.8763	0.0447	0.4025	−0.0662	0.1548	−0.228
	Docosahexaenoic acid	HMDB0002183	0.5184	−0.031	0.455	−0.0243	0.0093	−0.4204
	**Docosapentaenoic acid (22n-6)**	HMDB0001976	0.0707	0.1497	0.0147	0.0366	0.0036	0.1901
	Docosapentaenoic acid	HMDB0006528	0.0305	−0.322	0.1935	−0.231	0.0369	−0.3814
	Eicosapentaenoic acid	HMDB0001999	0.2179	0.0917	0.0423	0.1122	0.0051	−0.4765
	**Linoleic acid**	HMDB0000673	0.0077	0.3157	0.0317	0.2491	<1E-05	0.6928
	**Gamma-Linolenic acid**	HMDB0003073	0.0065	0.2699	0.0111	0.1437	0.001	0.5066
	**Alpha-Linolenic acid**	HMDB0001388	0.0065	0.2699	0.0111	0.1437	0.001	0.5066
	Stearidonic acid	HMDB0006547	1.00E-04	0.5806	0.0026	0.3783	0.3219	0.2299
Betaine metabolism	**5-Methyltetrahydrofolic acid**	HMDB0001396	<1E-05	0.5152	0.0073	0.4288	<1E-05	0.7356
	**Adenosine**	HMDB0000050	<1E-05	0.5206	2.00E-04	0.5026	<1E-05	0.767
	**Betaine**	HMDB0000043	2.00E-04	−0.6128	2.00E-04	−0.6276	4.00E-04	−0.6076
	Choline	HMDB0000097	0.149	0.246	0.1483	0.248	2.00E-04	0.4782
	**Dimethylglycine**	HMDB0000092	0.0099	−0.4097	2.00E-04	−0.5482	0.0349	−0.3979
	**FAD**	HMDB0001248	1.00E-04	0.4475	0.0012	0.3079	<1E-05	0.741
	**Homocysteine**	HMDB0000742	1.00E-04	0.5797	<1E-05	0.7265	0.0091	0.2224
	**L-Methionine**	HMDB0000696	0.0025	0.5037	3.00E-04	0.6065	1.00E-04	0.6249
	**NAD**	HMDB0000902	<1E-05	0.559	<1E-05	0.4802	<1E-05	0.7809
	**Phosphate**	HMDB0001429	5.00E-04	0.5015	1.00E-04	0.481	3.00E-04	0.6007
	**S-Adenosylhomocysteine**	HMDB0000939	<1E-05	0.6725	<1E-05	0.5406	<1E-05	0.6847
	S-Adenosylmethionine	HMDB0001185	0.4032	−0.1736	0.0439	−0.3987	<1E-05	−0.6663

*Metabolites with consistent results across batches and p < 0.10 are in bold*.

* *Measured metabolites in the 30 associated drug action pathways are the same*.

## Discussion

This study set out to investigate age-specific patterns of cellular metabolites in liver between birth and 18 years of age that may be indicative of altered drug response or susceptibility to drug toxicity unique to pediatric patient populations. Using an untargeted mass-spectroscopy based metabolomics analysis; we assessed hundreds of metabolites in a set of pediatric liver samples, with both technical and biological validation built into the study design (Figure 1A). A major challenge for investigations like the one we have conducted is access to a large number of tissue samples of sufficiently high quality to obtain interpretable results; differences in retrieval and preservation methods across various publicly funded and commercial providers has considerable potential to not only contribute to variability in sample quality (16), but may also confound data interpretation. For example, in the United States, availability of tissue samples from infants <1 year of age is relatively infrequent, and those that are available for research tend to be procured from the NIH-funded University of Maryland Brain and Tissue Bank for Developmental Disorders. This period of time also tends to be a period of rapid growth and can be accompanied by developmental patterns of gene expression as we noted in a previous study involving RNA-Seq analysis of a similar set of samples (17). Thus, it is difficult to differentiate true developmental differences in RNA or metabolite expression from confounding effects related to tissue source. Given that initial statistical analysis of the metabolomic data corroborated the source effect in the RNA-Seq data (17), the current investigation included samples <1 year of age from a second, independent source.

This experimental approach detected 24 metabolites that changed in a set of liver samples spanning birth to late adolescence (18 years of age; Table 2). Of the 24 metabolites, 21 were found to have increasing levels over childhood development with the lowest levels present in the infant age group (children <1 year of age) and little difference in metabolite levels between the three older age groups (children >1 year of age) (Figure 3 and Supplemental Figure 1). Key sub-pathways were observed in the list of significant known metabolites, including “purine metabolism (hypo)xanthine/Inosine containing,” “pyrimidine metabolism, uracil containing,” and “long fatty acids”—all three replicated metabolites in long fatty acids sub-pathway increasing in abundance throughout childhood development (Table 2). Lastly, we observed consistent age-related differences in KEGG pathways “Betaine Metabolism” (pathway *p*-value of < 1.0 × 10^−5^ for all 3 batches) and “Alpha-Linolenic Acid and Linoleic Acid Metabolism” (pathway *p*-value of 0.002, 0.007, and < 1.0 × 10^−5^ for batch 1, batch 2 and batch 3, respectively). A limitation to our analysis is that metabolites may not be considered simply because their concentration is natural concentration is near the lower limit of detection. There were 353 metabolites that were measured in all three batches and of these 31 metabolites were removed because there was 75% missing in at least one batch (Supplemental Figure 3). Interestingly, X – 11,795, glycerol 2–phosphate, gamma–glutamylhistidine, formiminoglutamate, and disulfide, benzoate, may have been removed due to the different technologies being used in Batch 1, and Batches 2 and 3. On the other hand, many of the other metabolites have a consistently high percentage of missing observations across all batches (Supplemental Figure 4).

Individual analytes and those assigned to specific pathways may be derived from both endogenous as well as exogenous (dietary) sources. For example, the betaine metabolism pathway contained 12 measured metabolites, with all but two metabolites found to be related to ontogeny (*p* < 0.10). Eight out of the 10 ontogeny related metabolites increased in abundance increased with age (Table 3 and Figure 4A). In humans, betaine itself is found in food and can also be formed endogenously from choline. Betaine is vital in transmethylation and provides control of hepatocellular hydration and provides protection of the liver from various forms of stress, including osmotic stress (18). Similarly, within the “Alpha-Linolenic Acid and Linoleic Acid Metabolism” pathway, 4 metabolites consistently were observed to increase with increasing age across the batches with *p* < 0.10: docosapentaenoic acid, docosapentaenoic acid (22n-6), linoleic acid, gamma-linolenic acid and alpha-linolenic acid, whereas arachidonic acid was present at higher concentrations in children <1 year of age relative to the older age groups (Table 3 and Figure 4B). Alpha-linolenic acid (ALA) is an essential omega-3 fatty acid found in many nuts and vegetable oils, and thus it is difficult to differentiate analyte changes due to biological maturation with external factors that also change with increasing age.

One obvious difference between infants <1 year of age and older children/adolescents is diet, with breast milk and formula, either cow's milk- or soy-based formulas, representing the primary source of nutrition until solid oral foods are introduced later in the first year of life. However, it is also now well-recognized that the intestinal microbiota of newborns is different from older children and adults, being established after birth and influenced not only by mode-of delivery, vaginal vs. cesarean, but also to a considerable extent by the mode of feeding. For example, several studies now report higher levels of *Lactobacilli* and bifidobacteria in stool from breast-fed infants compared to stool from formula-fed infants that is dominated by a more diverse variety of species, Bacteroides, Clostridia, Staphylococci, enterobacteria, *Enterococci*, and Atopobium (19–21). Introduction of solid foods also results in increased diversity of the intestinal microbiota over time. Thus, it is not unreasonable to expect that concentrations of dietary constituents and the products of metabolism of those constituents by a changing intestinal microbiota might result in a unique infant metabolome.

Developmental differences in the gut microbiome between infants <1 year of age and older children, or more specifically establishment of the gut microbiome after birth, would be expected to result in changes in bile acid composition. For example, primary bile acids derived from endogenous liver metabolism would be expected to be higher and the concentrations of gut microbiome-derived secondary bile acids to be lower in the younger group, with a shift toward accumulation of predominantly secondary bile acids in the older age groups following maturation of the intestinal microbiome. Higher concentrations of primary bile acids in premature infants and term newborns compared to older ages is a well-known phenomenon (22, 23), and our observation of lower concentrations of primary and secondary bile acids taurodeoxycholate and glycolithocholate sulfate in the <1 year old group is consistent with colonization and maturation of the gut microbiome over the first year of life.

Studies in which germ-free mice are transferred to a conventional environment simulate the change in environment experienced during birth when newborns enter their extrauterine environment. Claus et al. (24) applied metabolomic approaches to characterize the metabolic adaptation to bacterial colonization of the gut by transferring germ-free mice to conventional environment in which bedding used by conventional mice was provided to expose the germ-free animals to the same microbial ecosystem. They observed rapid increases in weight over the first few days after colonization as well as increases in gluconeogenesis that were followed by increases in hepatic triglyceride synthesis and alterations in bile acid metabolites. It is interesting to note that five analytes in our dataset that were differentially present between the <1 year old and older age groups, heme, taurodexycholate, 10-heptadecenoate (17:1n7), 10-non-adecenoate (19:1n9), and margarate (17:0), were also observed to change by Claus et al., supporting the hypothesis that some of the observed “developmental” changes in metabolome are a consequence of bacterial colonization of the gut after birth.

In conclusion, this limited dataset illustrates the complexity underlying observed “developmental” changes in the hepatic metabolome as assessed by the snapshot of developmental changes provided by metabolomic analysis of liver tissue. The first year of life is characterized by a velocity of change in height and weight that exceeds that observed in older children, and thus, some changes in the metabolome reflect the processes of growth and development. However, it is apparent that other factors, such as change in diet and gut microbiome with increasing age, also contribute to the overall picture. The interplay between diet, gut microbiome and hepatic physiology can be expected to underlie developmental changes in expression of drug metabolizing enzymes and transporters involved in the absorption, distribution, metabolism and excretion of drugs, nutrients, toxicants, and other foreign compounds. For example, bile salts are essential for absorption of lipid soluble vitamins and poorly water-soluble medications. Furthermore, expansion of the bile acid pool is also accompanied by changes in expression of hepatocellular and biliary uptake and efflux transporters (25) and likely contributes to the developmental trajectories of transporters that is of considerable interest for modeling and simulation of drug disposition in pediatrics (26). Studies with germ-free mice introduced into a conventional environment also resulted in increased expression of drug metabolizing enzymes, such as CYP2C29 and CYP3A11 that are similar to the developmental trajectories of important drug biotransformation pathways in humans, such as CYP2C9 (27) and CYP3A4 (28). Thus, improved understanding of the factors contributing to the developmental processes governing the role of the liver and its interaction with other systems will improve our understanding of not only drug disposition in a vulnerable, understudied patient population, but will also aid in identification of factors that influence drug disposition and response in individual children.

## Data Availability Statement

The normalized and imputed data used in the analysis along with the analysis results can be found at http://explorerpedpgx.moffitt.org using Google Chrome. The data after QC is also available for download on the Metabolomic workbench, https://www.metabolomicsworkbench.org/. These data can be found with the following study numbers are ST001402, ST001403, and ST001404 corresponding to Batch 1, 2, 3 respectively.

## Ethics Statement

The studies involving human participants were reviewed and approved by the use of these tissues was classified as non-human subject research by the Children's Mercy Hospital Pediatric Institutional Review Board. A replication set of post-mortem liver tissue samples from autopsies of fetuses (from therapeutic abortions or stillbirths) and infants was provided by the Erasmus Medical Center Tissue Bank, Sophia Children's Hospital, Rotterdam, the Netherlands. The Erasmus Medical Center Research Ethics Board waived the need for formal ethics approval according to the Dutch Law on Medical Research in Humans. Tissue was collected when parental written informed consent for both autopsy and the explicit use of the tissue for research was present. Written informed consent to participate in this study was provided by the participants' legal guardian/next of kin.

## Author Contributions

SW and JL conceived and planned the experiments. CW, QL, CB, and BF completed the statistical and bioinformatics analysis. JL and BF supervised the project. RG, SW, JL, and CB completed experiments. CW, JL, and BF wrote the manuscript. All authors reviewed and edited the manuscript.

## Conflict of Interest

The authors declare that the research was conducted in the absence of any commercial or financial relationships that could be construed as a potential conflict of interest.

## Supplementary Material

Supplemental Figure 1 Side by side boxplots of metabolite concentration and age for the 25 biologically and technically validated metabolites for each of the three batches.

Supplemental Figure 2 Drug action pathways. The results for metabolites measured in this study are indicated with red indicating the metabolite was increasing with age and blue indicating the metabolite was decreasing in abundance with age. Metabolites in the pathway not measured are indicated in black. The shape corresponds to the significance of the Spearman correlation of metabolite's concentration and age, where a square indicates that the *p* < 0.05 and a circle shows that the *p* > 0.05. For the drug action pathway, the one individual metabolite that is unique to a drug action pathway is denoted by a purple trapezoid.

Supplemental Figure 3 Venn diagram of the number of metabolites removed from analysis, by batch, because 75% of the observations were missing in at least one batch (31 metabolites).

Supplemental Figure 4 Heatmap of the percent of observations missing for metabolites that were eliminated from analysis due to more than 75% of observations were missing for at most 2 batches (20 metabolites).

Supplemental Table 1 Results for all 905 metabolites in batch 2. N infant = 13, N early childhood = 14, N late childhood = 10, N adolescent = 11.

Supplemental Table 2 Results for all 914 metabolites in batch 3. N infant = 21, N early childhood = 7, N late childhood = 11, N adolescent = 11.

Supplemental Table 3 Results for all 663 metabolites in batch 1. N infant = 13, N early childhood = 14, N late childhood = 10, N adolescent = 11.

Supplemental Table 4 Results for all 32 KEGG and drug pathways with at least 5 metabolites and pathway coverage of at least 50%.
