# Supplementary figures and images for: Ontogeny Related Changes in the Pediatric Liver Metabolome
Source: Front Pediatr. 2020 Sep 29;8:549. doi: 10.3389/fped.2020.00549 (PMC7550739; doi:10.3389/fped.2020.00549)

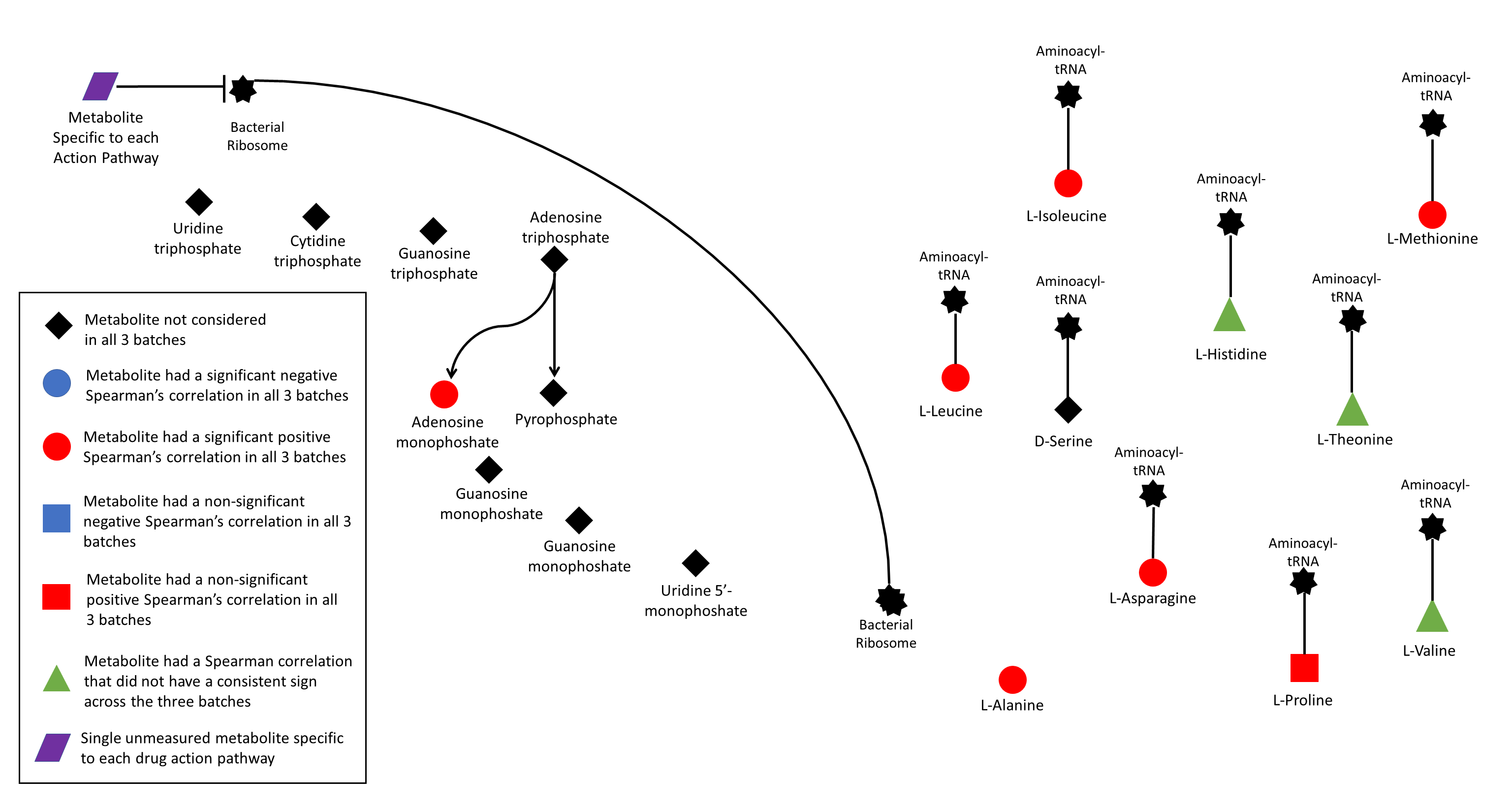

Supplement: Supplemental Figure 1 — Side by side boxplots of metabolite concentration and age for the 25 biologically and technically validated metabolites for each of the three batches. [file Image_1.TIF]

Batch1

Batch2

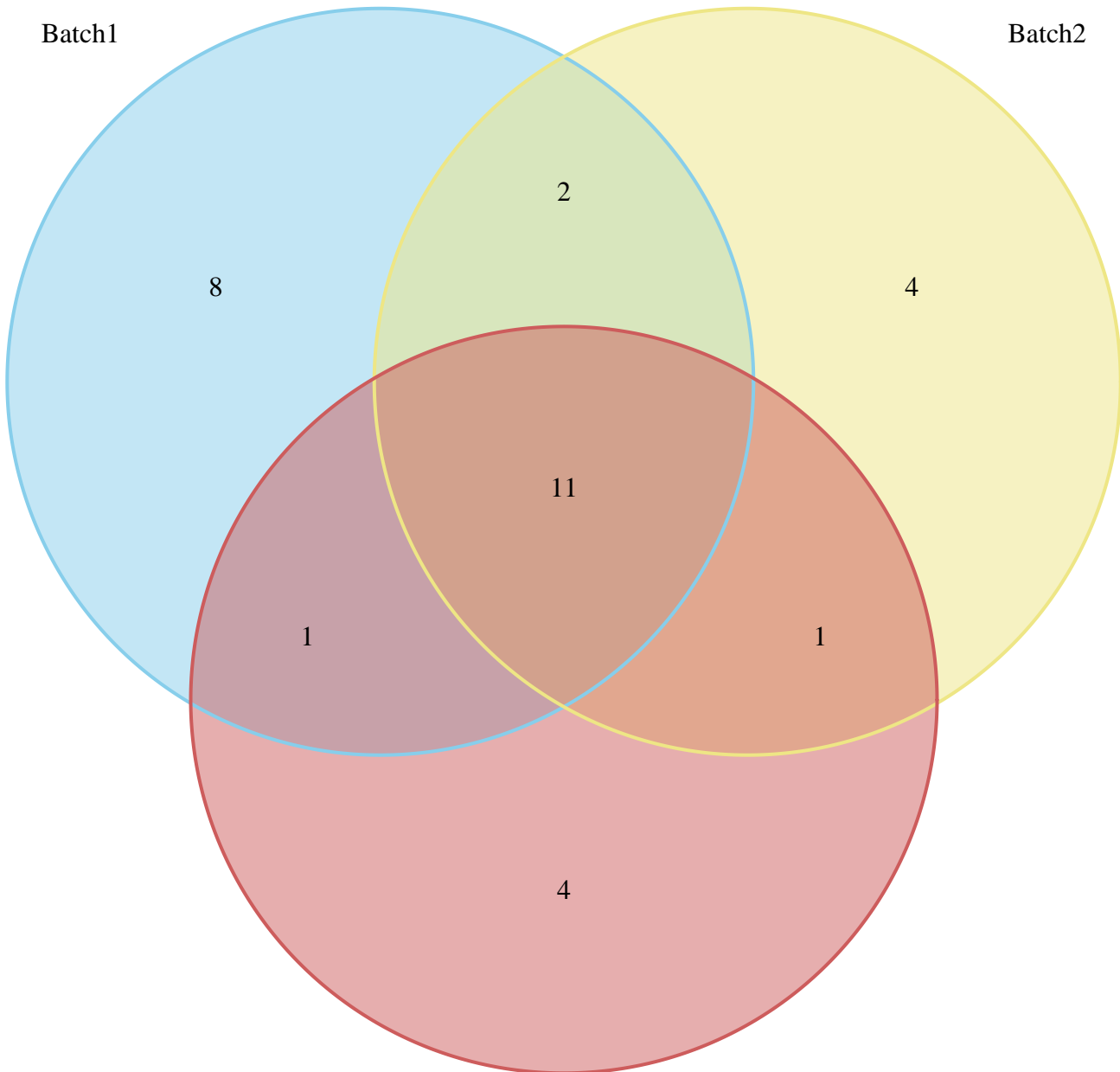

Batch3

Supplement: Supplemental Figure 3 — Venn diagram of the number of metabolites removed from analysis, by batch, because 75% of the observations were missing in at least one batch (31 metabolites). [file Data_Sheet_2.PDF]

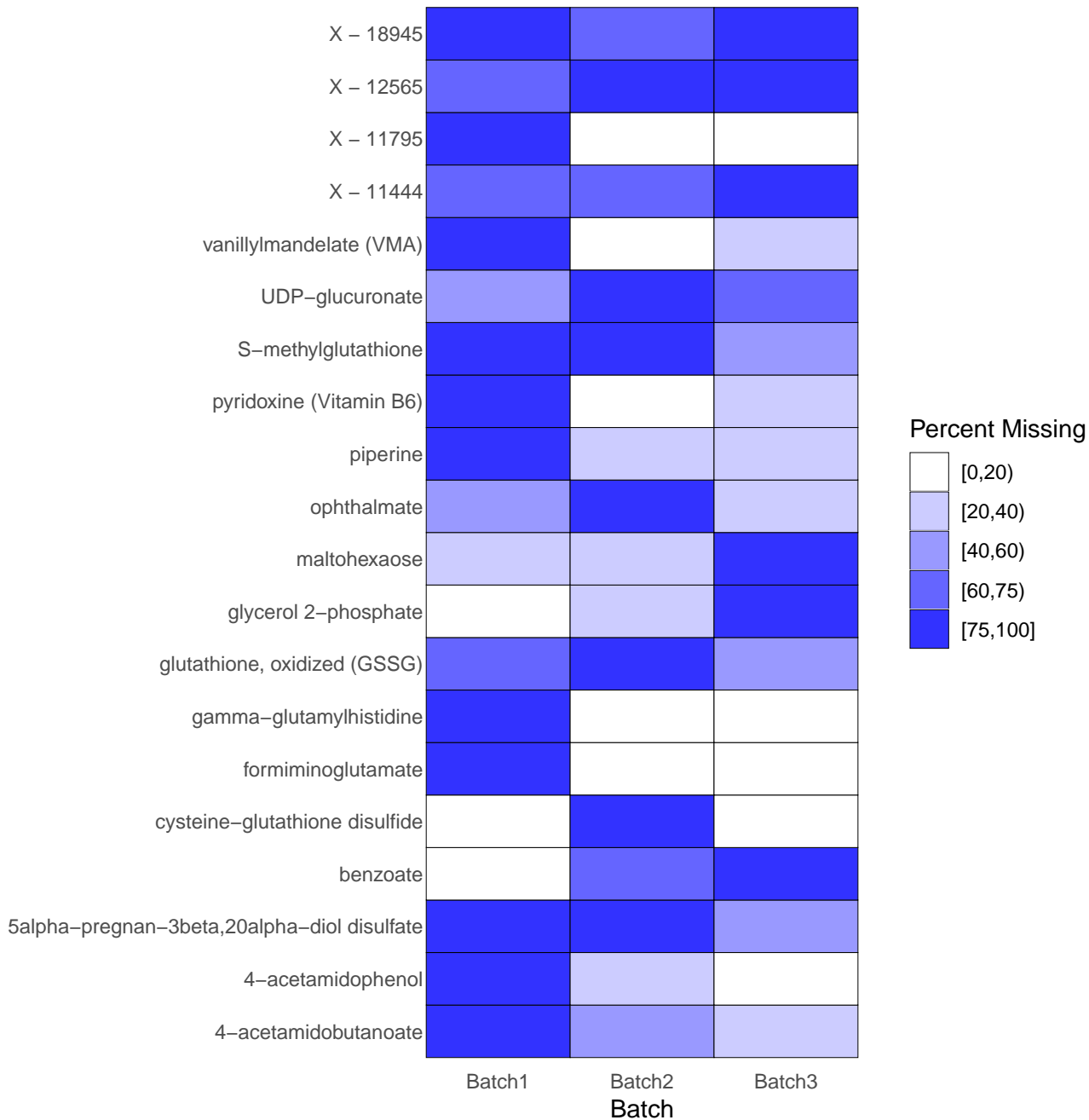

Supplement: Supplemental Figure 4 — Heatmap of the percent of observations missing for metabolites that were eliminated from analysis due to more than 75% of observations were missing for at most 2 batches (20 metabolites). [file Data_Sheet_3.PDF]
