# Supplementary material for: Ontogeny Related Changes in the Pediatric Liver Metabolome
Source: Front Pediatr. 2020 Sep 29;8:549. doi: 10.3389/fped.2020.00549 (PMC7550739; doi:10.3389/fped.2020.00549)
Supplement: Supplemental Figure 2 — Drug action pathways. The results for metabolites measured in this study are indicated with red indicating the metabolite was increasing with age and blue indicating the metabolite was decreasing in abundance with age. Metabolites in the pathway not measured are indicated in black. The shape corresponds to the significance of the Spearman correlation of metabolite's concentration and age, where a square indicates that the p < 0.05 and a circle shows that the p > 0.05. For the drug action pathway, the one individual metabolite that is unique to a drug action pathway is denoted by a purple trapezoid. [file Data_Sheet_1.PDF]

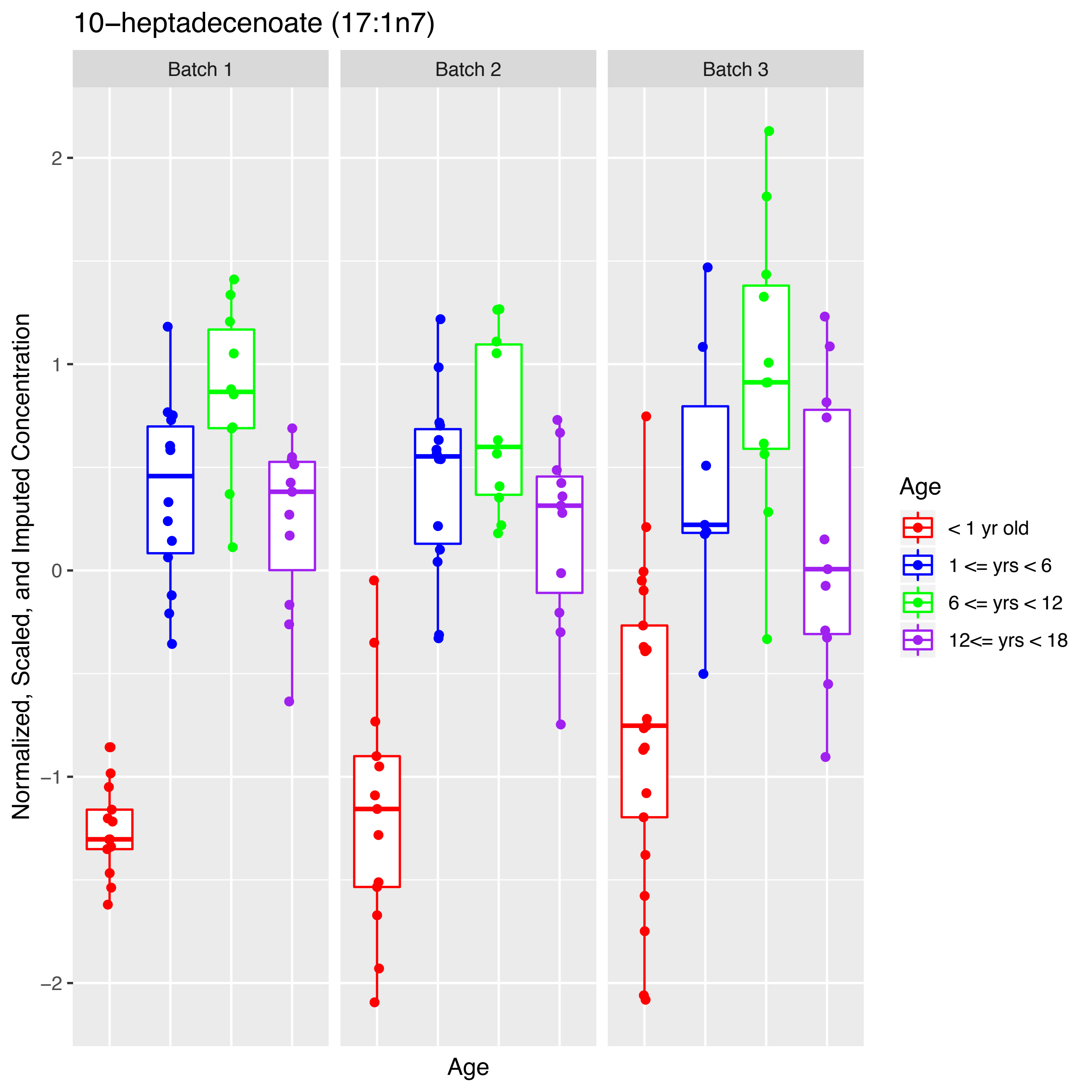
10−heptadecenoate (17:1n7)
Batch 1
Batch 2
Batch 3
2
1
0
−1
−2
Normalized, Scaled, and Imputed Concentration
Age
Age
< 1 yr old
1 <= yrs < 6
6 <= yrs < 12
12<= yrs < 18

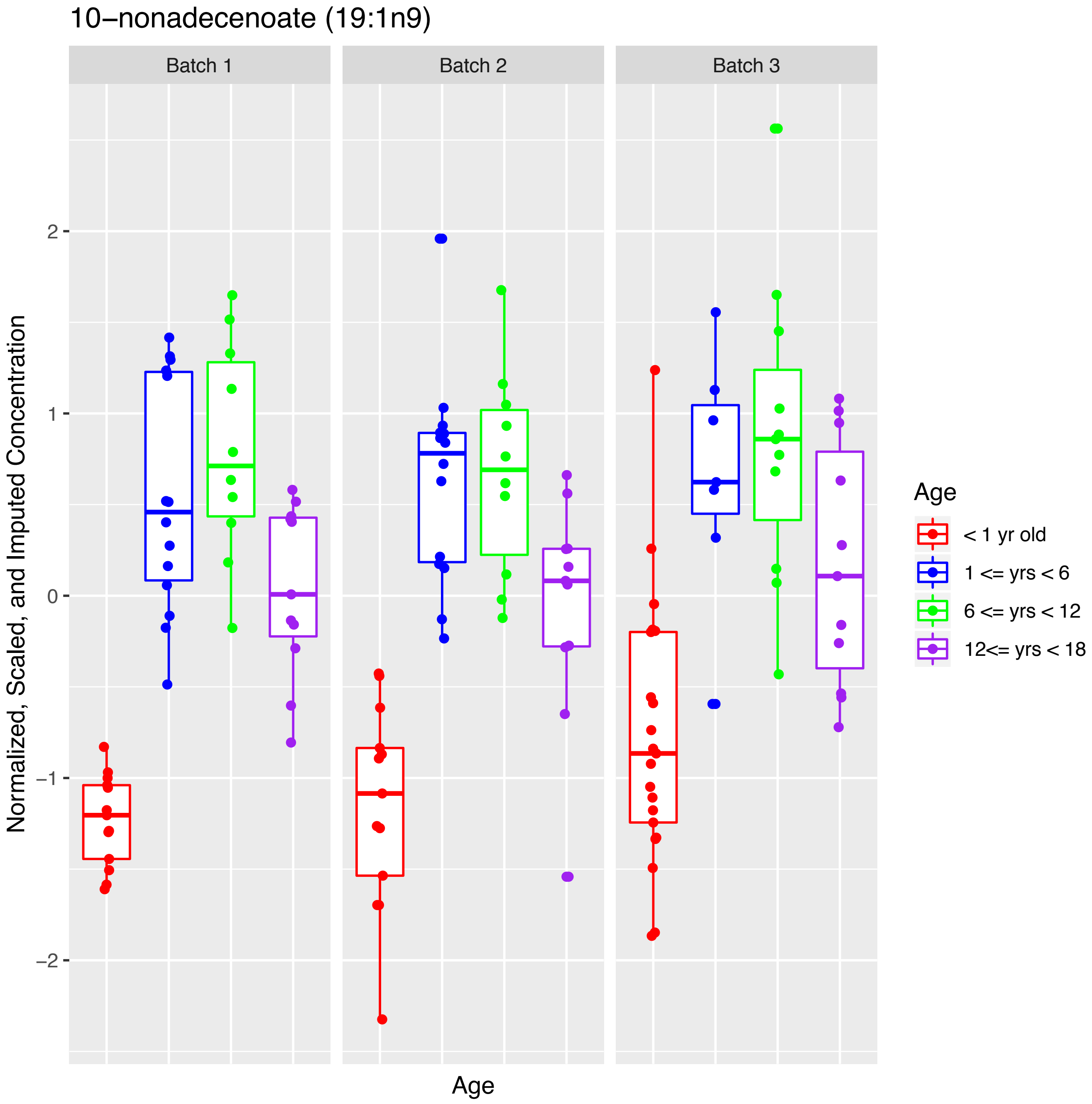

10−nonadecenoate (19:1n9)
Batch 1
Batch 2
Batch 3
Normalized, Scaled, and Imputed Concentration
2
1
0
−1
−2
Age
Age
< 1 yr old
1 <= yrs < 6
6 <= yrs < 12
12<= yrs < 18

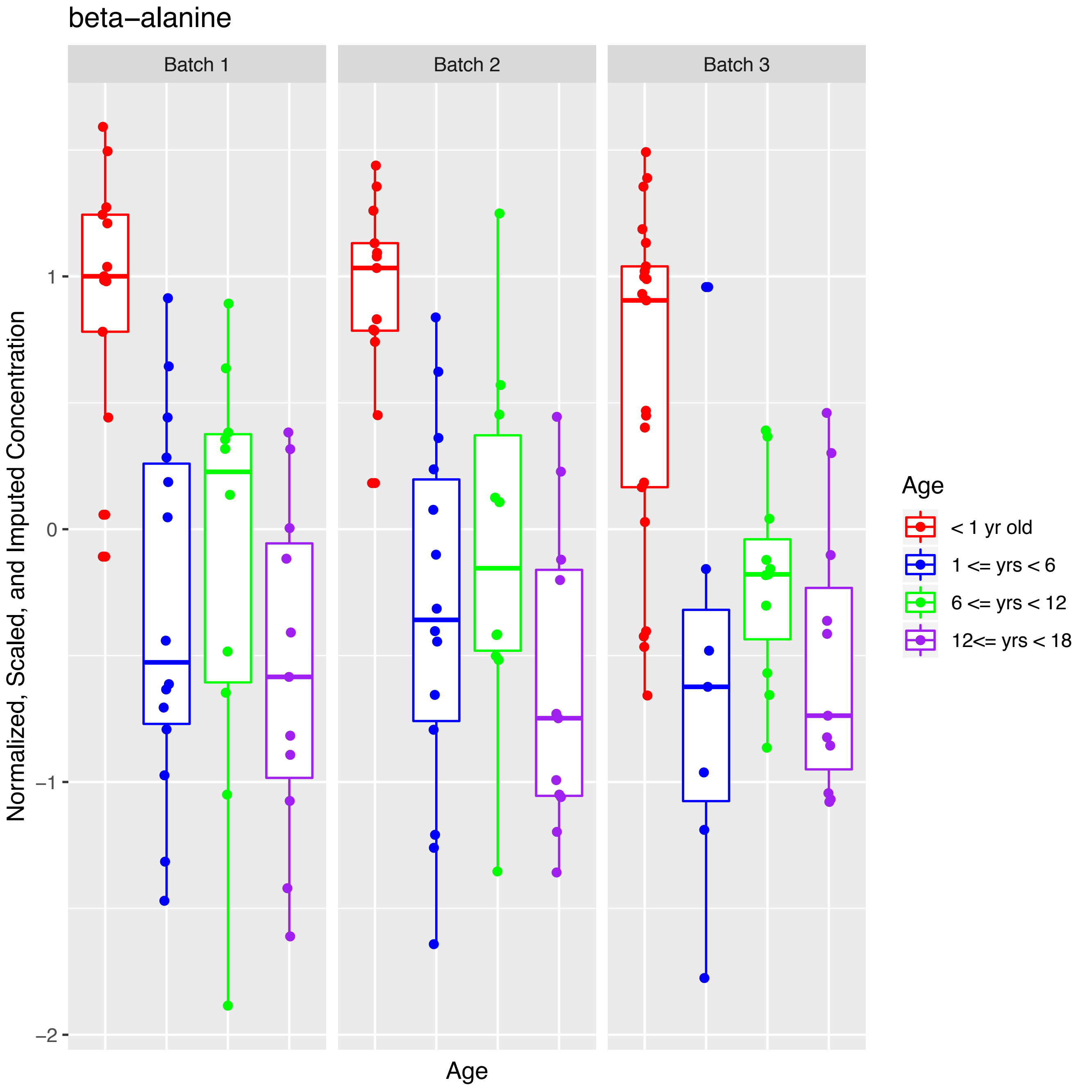
beta−alanine
Batch 1
Batch 2
Batch 3
1
0
−1
−2
Normalized, Scaled, and Imputed Concentration
Age
Age
< 1 yr old
1 <= yrs < 6
6 <= yrs < 12
12<= yrs < 18

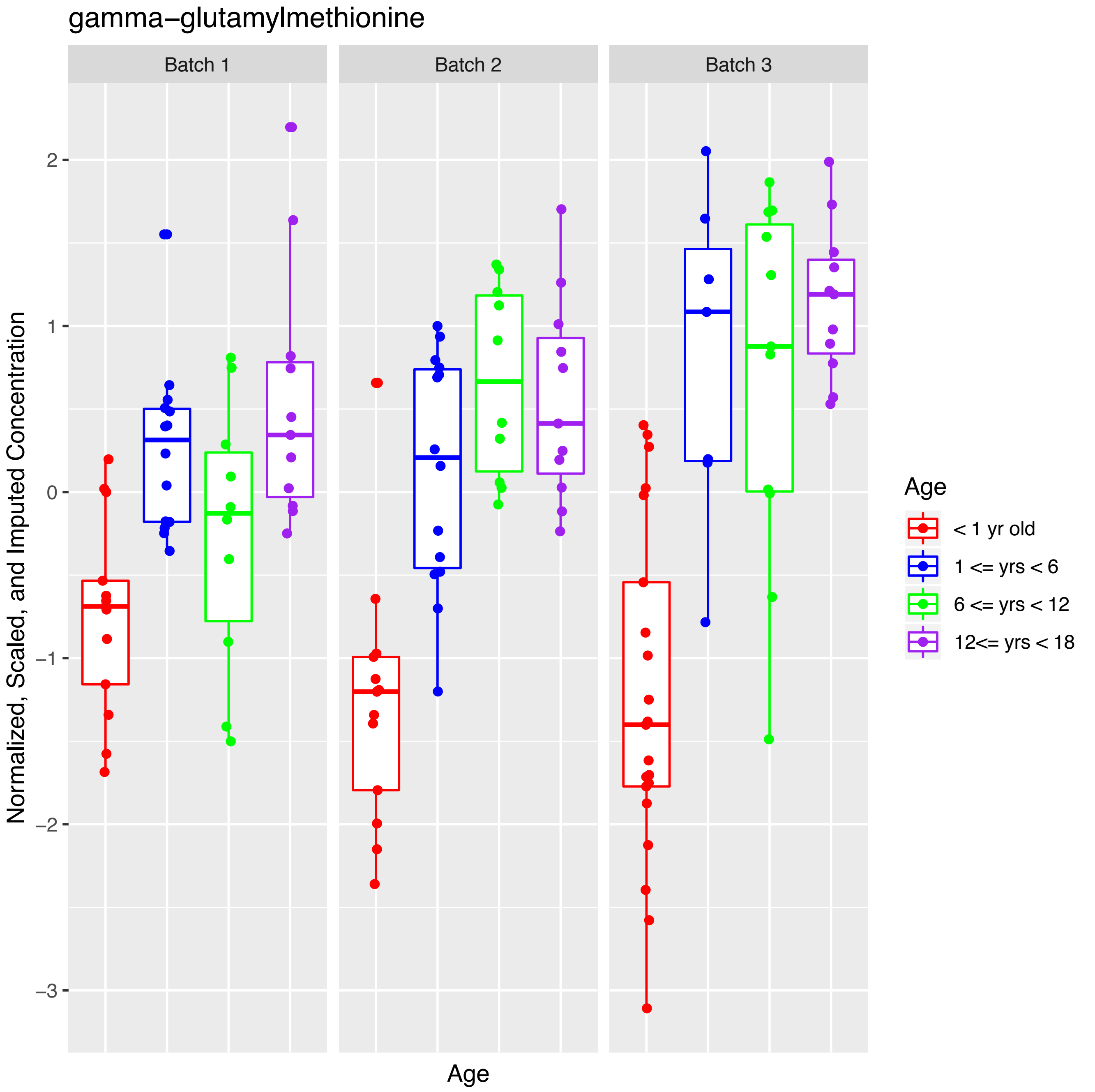
gamma−glutamylmethionine
Batch 1
Batch 2
Batch 3
Normalized, Scaled, and Imputed Concentration
2
1
0
−1
−2
−3
Age
Age
< 1 yr old
1 <= yrs < 6
6 <= yrs < 12
12<= yrs < 18

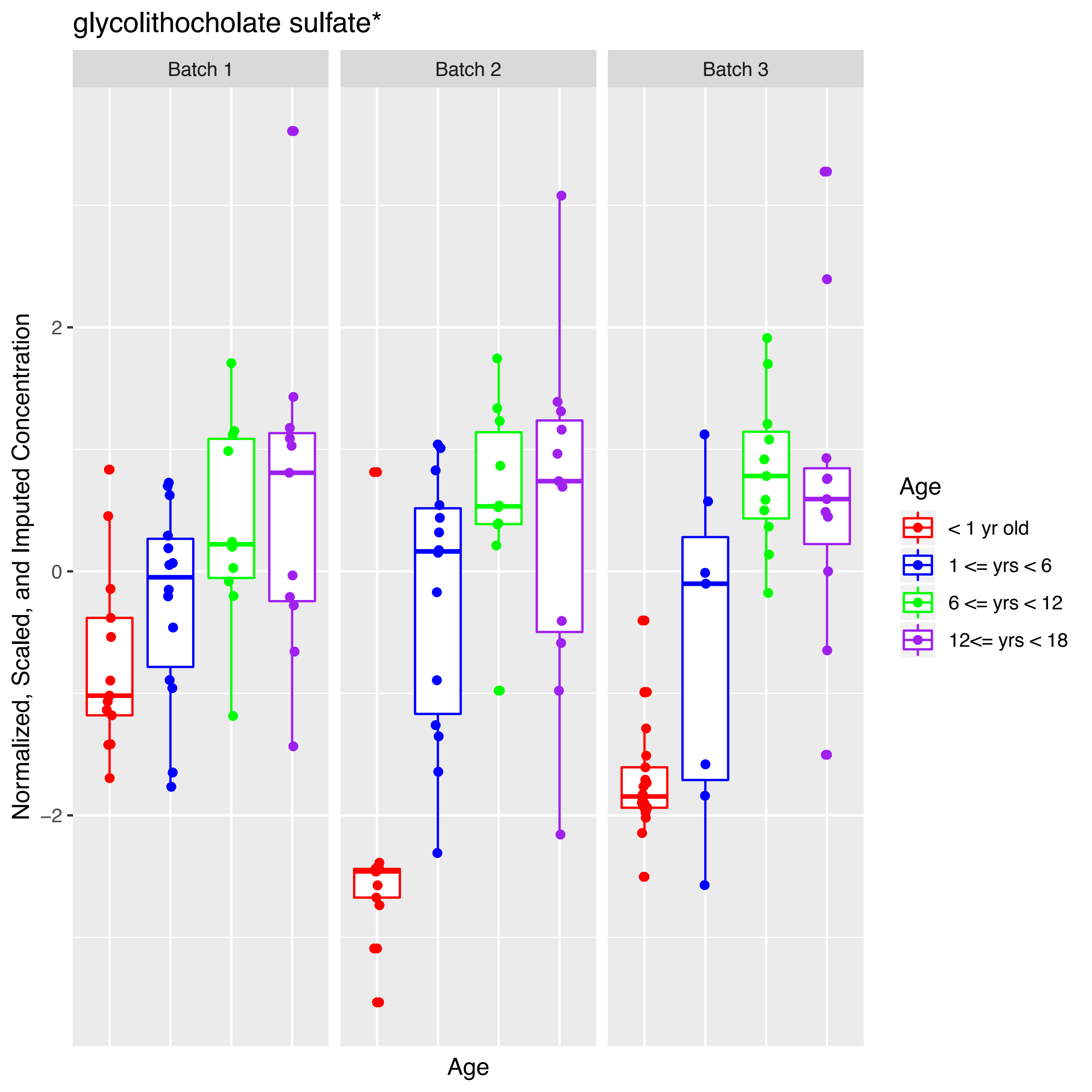

glycolithocholate sulfate*
Batch 1
Batch 2
Batch 3
Normalized, Scaled, and Imputed Concentration
2
0
−2
Age
Age
< 1 yr old
1 <= yrs < 6
6 <= yrs < 12
12<= yrs < 18

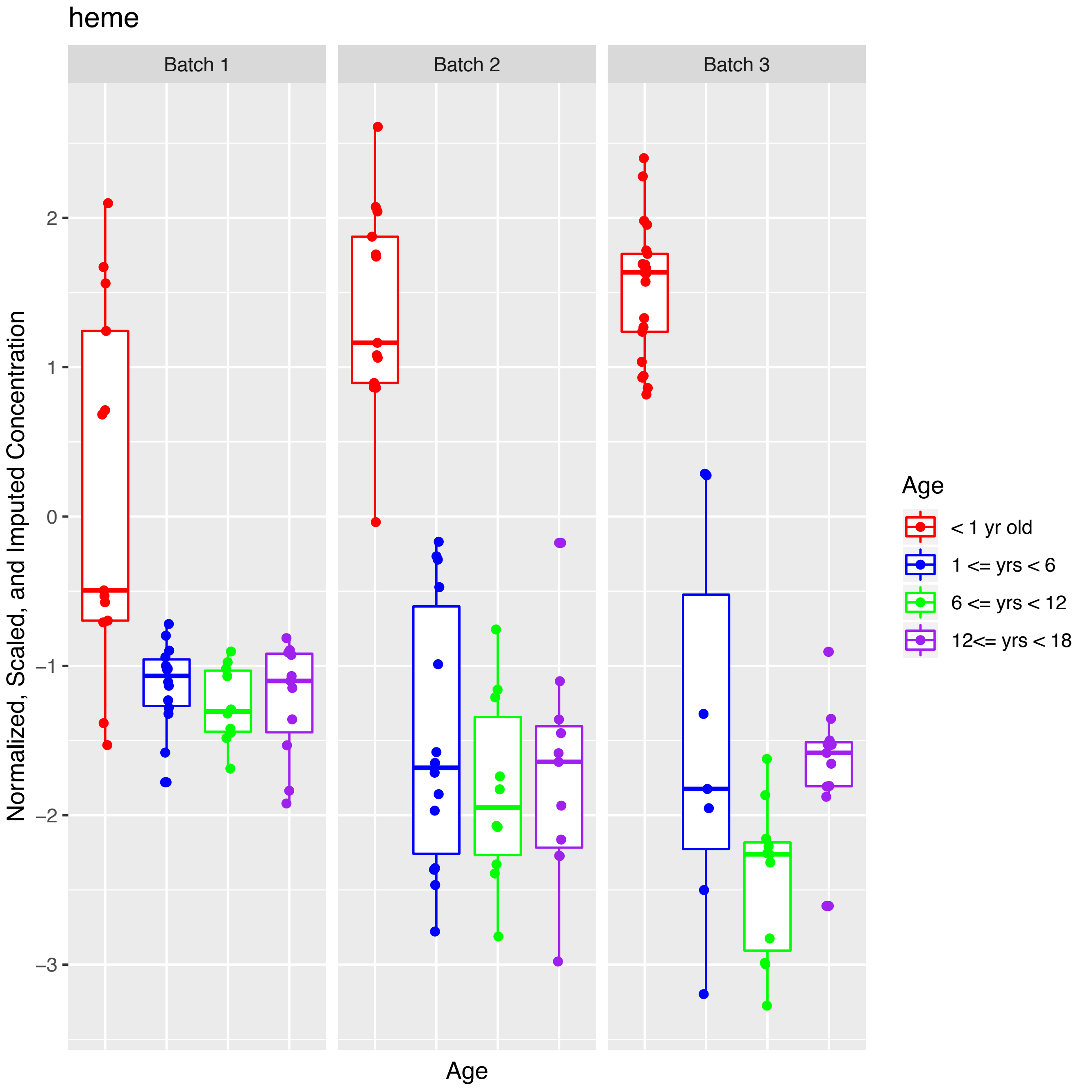
heme
Batch 1
Batch 2
Batch 3
Normalized, Scaled, and Imputed Concentration
2
1
0
−1
−2
−3
Age
Age
< 1 yr old
1 <= yrs < 6
6 <= yrs < 12
12<= yrs < 18

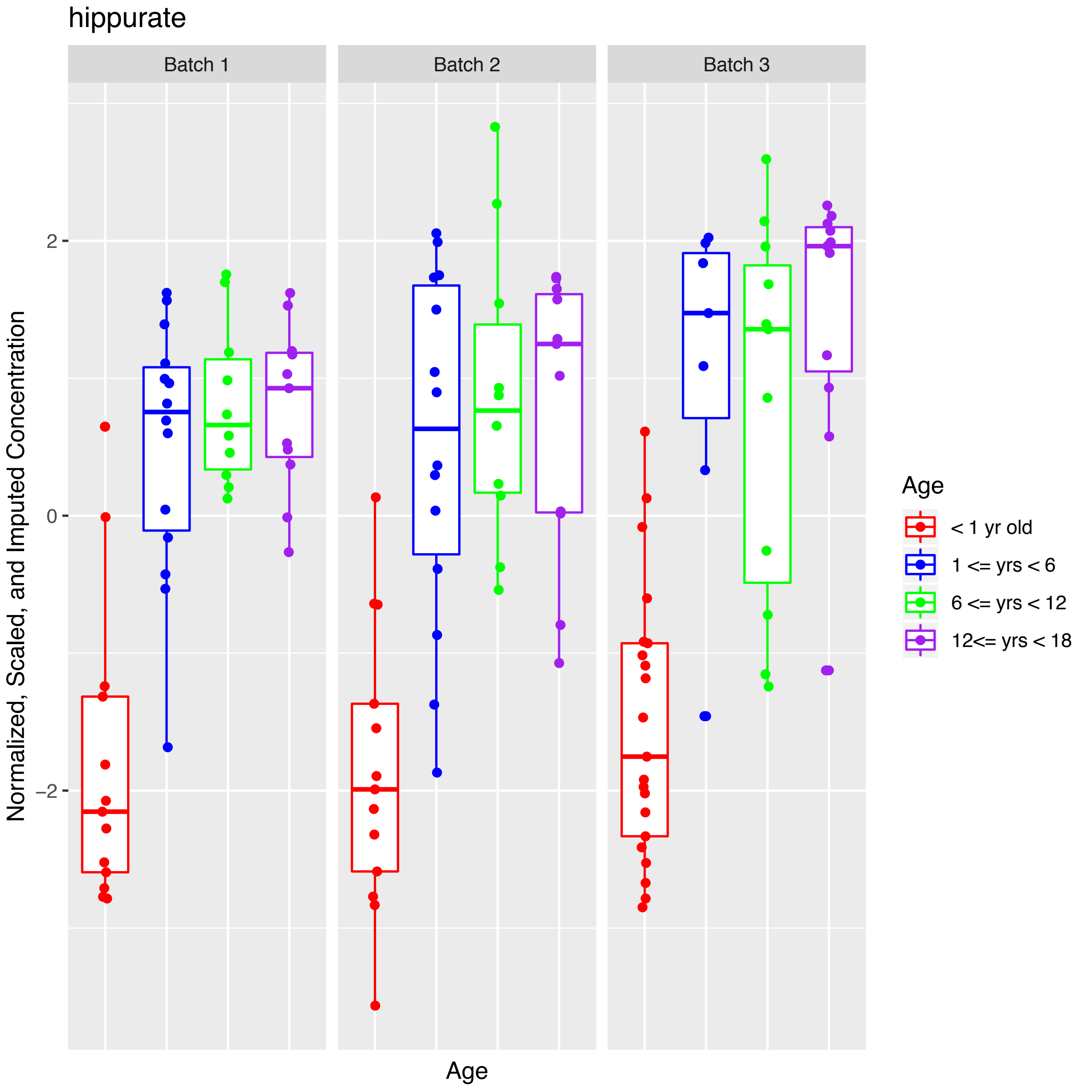

hippurate
Batch 1
Batch 2
Batch 3
2
0
−2
Normalized, Scaled, and Imputed Concentration
Age
Age
< 1 yr old
1 <= yrs < 6
6 <= yrs < 12
12<= yrs < 18

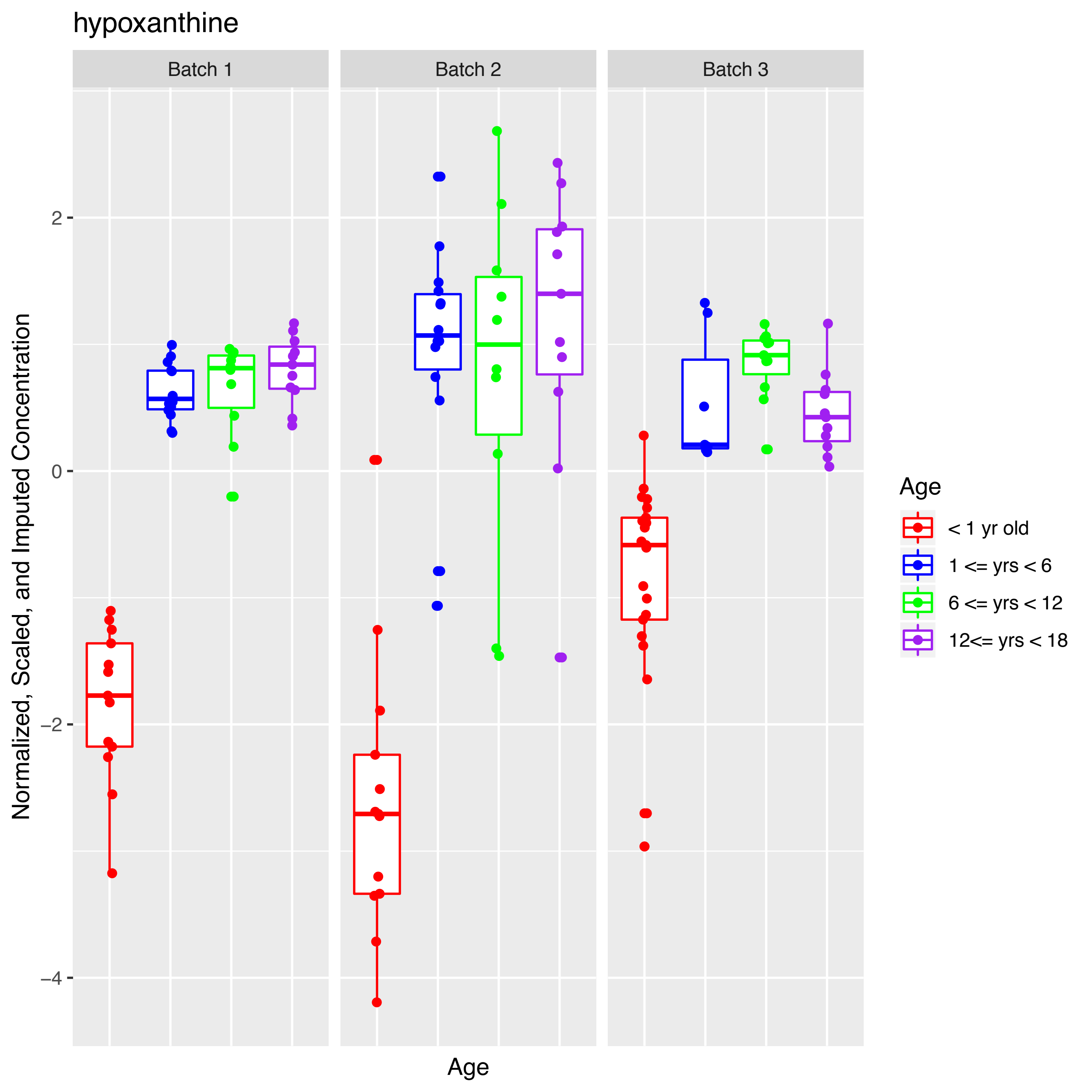

hypoxanthine
Batch 1
Batch 2
Batch 3
Normalized, Scaled, and Imputed Concentration
2
0
−2
−4
Age
Age
< 1 yr old
1 <= yrs < 6
6 <= yrs < 12
12<= yrs < 18

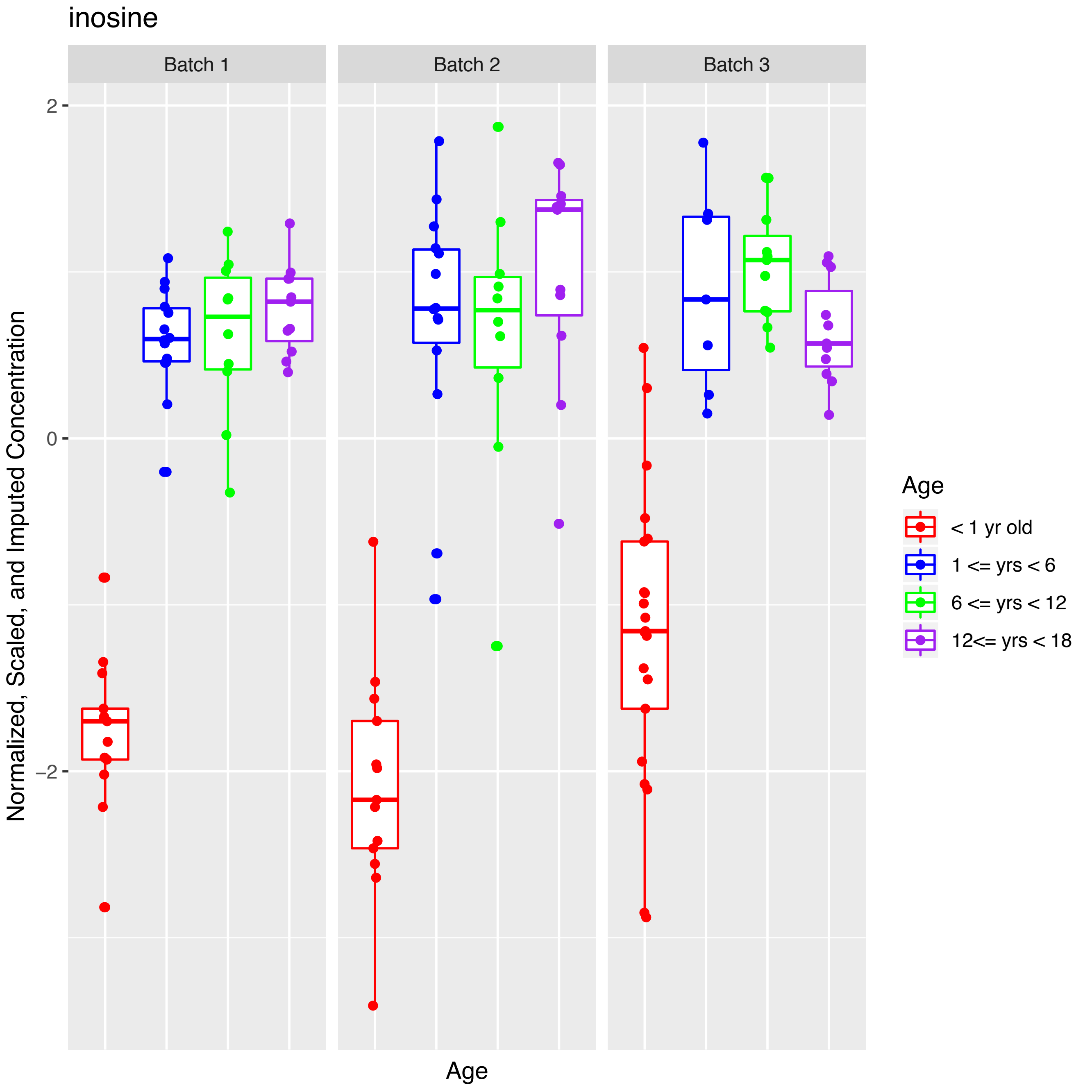

inosine
Batch 1
Batch 2
Batch 3
2
0
−2
Normalized, Scaled, and Imputed Concentration
Age
Age
< 1 yr old
1 <= yrs < 6
6 <= yrs < 12
12<= yrs < 18

lactobionate

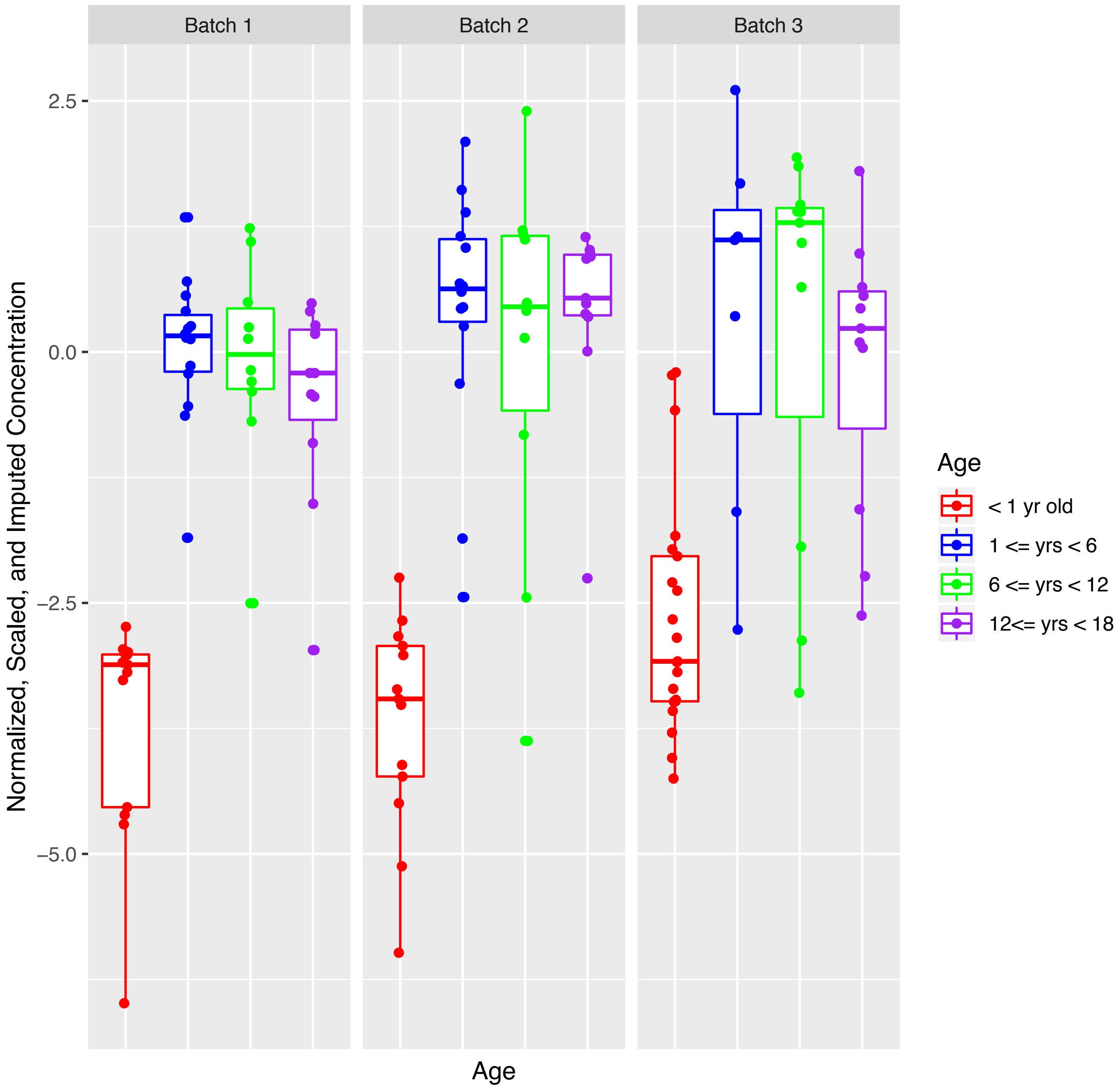

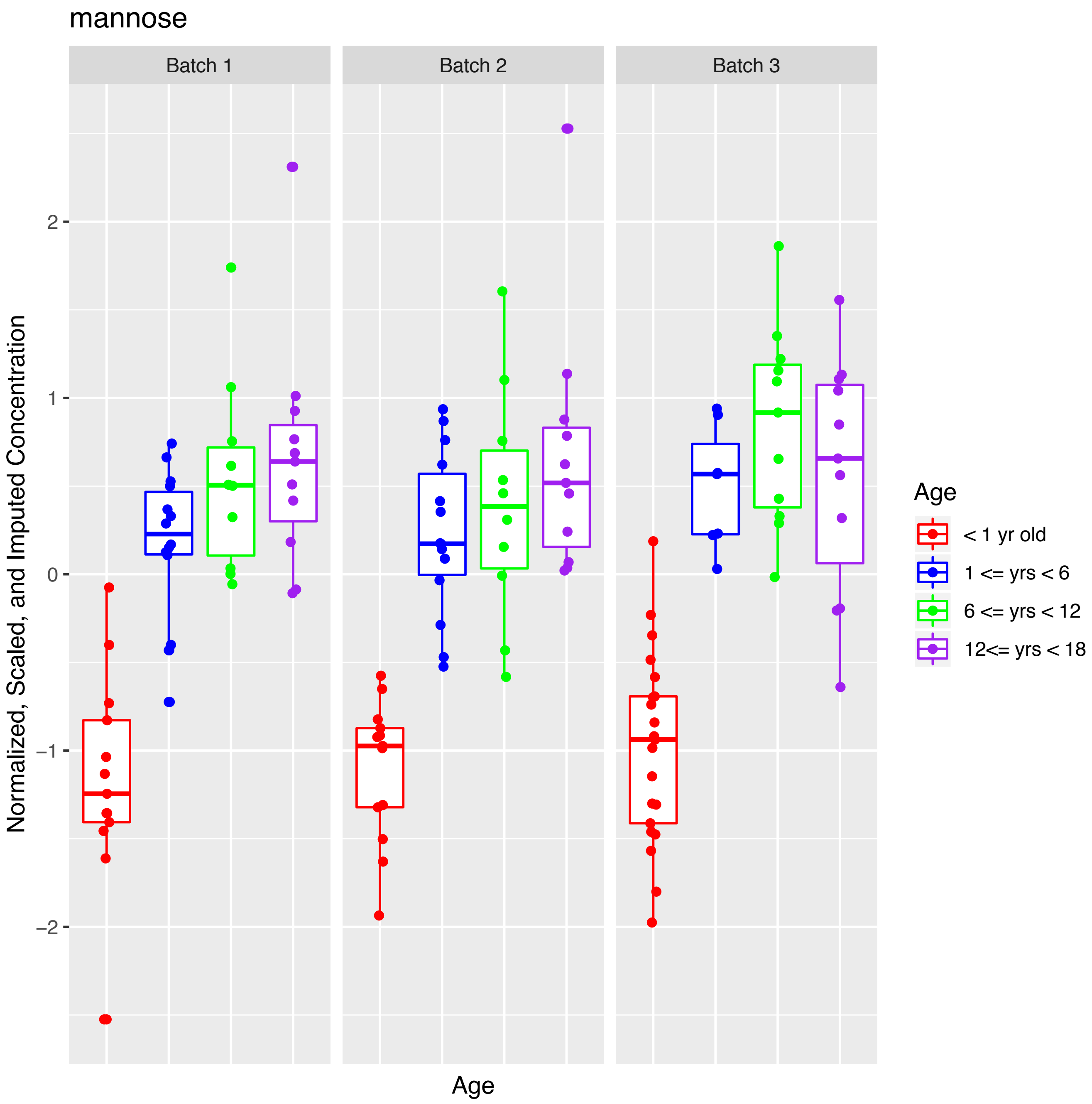
mannose
Batch 1
Batch 2
Batch 3
Normalized, Scaled, and Imputed Concentration
2
1
0
−1
−2
Age
Age
< 1 yr old
1 <= yrs < 6
6 <= yrs < 12
12<= yrs < 18

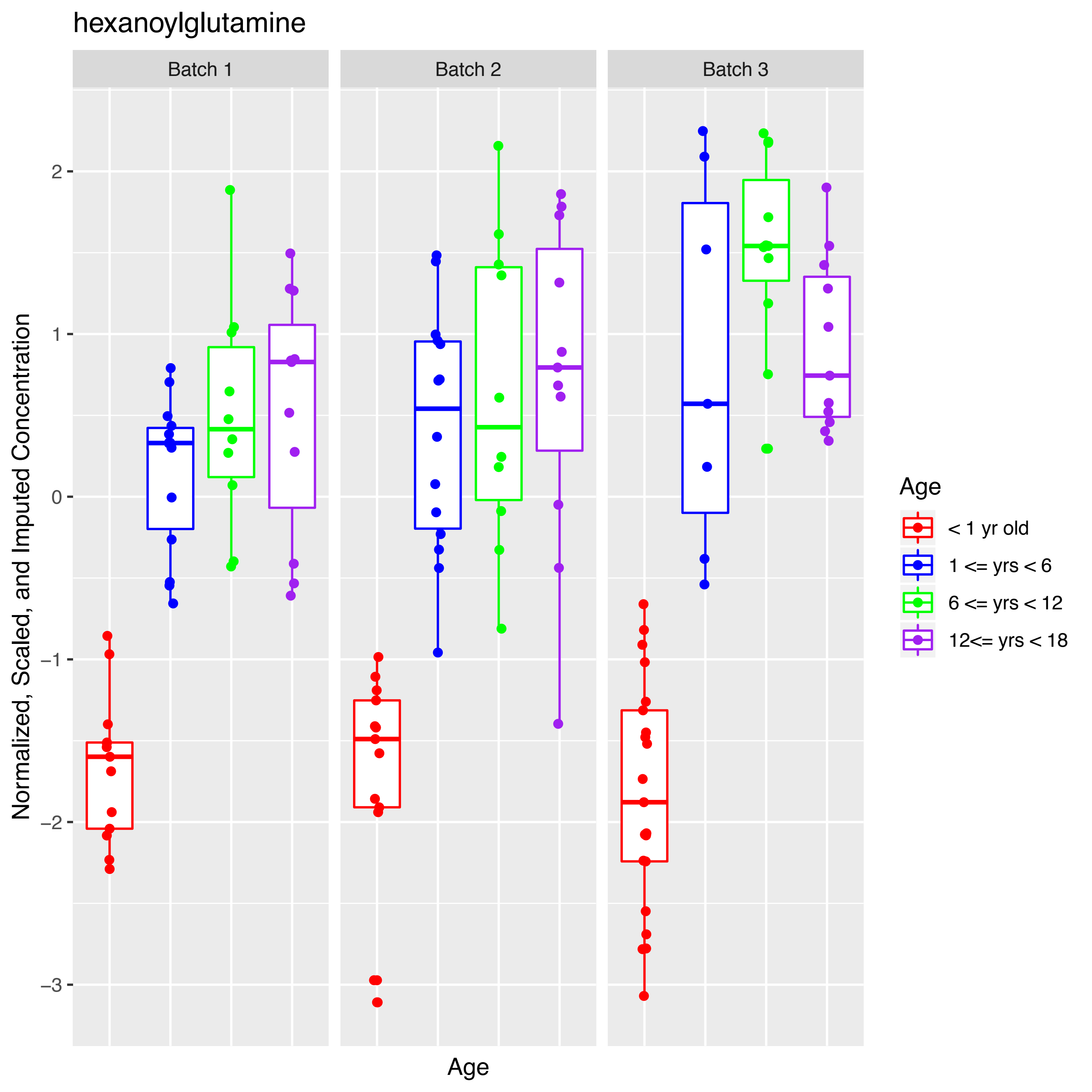

hexanoylglutamine
Batch 1
Batch 2
Batch 3
Normalized, Scaled, and Imputed Concentration
2
1
0
−1
−2
−3
Age
Age
< 1 yr old
1 <= yrs < 6
6 <= yrs < 12
12<= yrs < 18

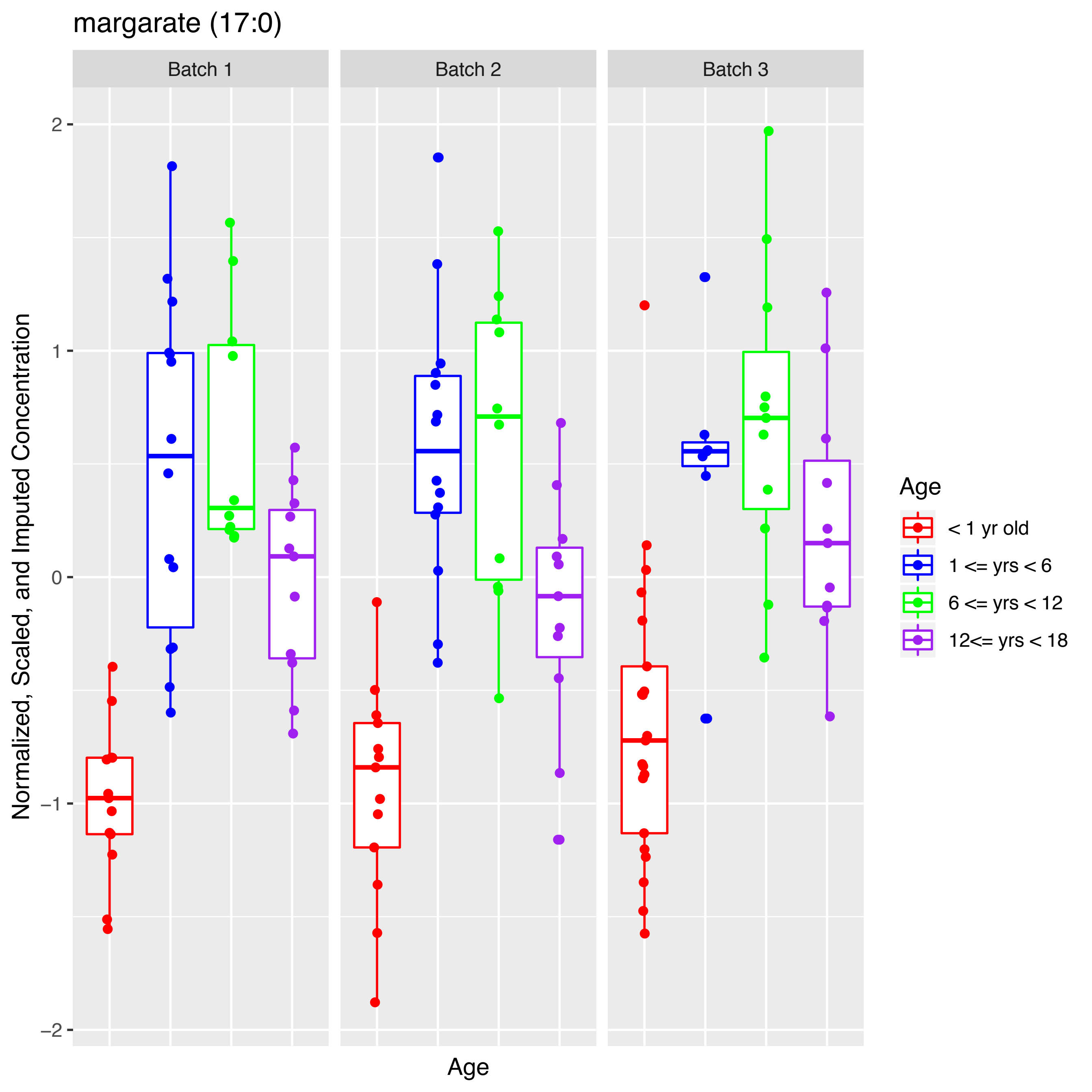
margarate (17:0)
Batch 1
Batch 2
Batch 3
Normalized, Scaled, and Imputed Concentration
2
1
0
−1
−2
Age
Age
< 1 yr old
1 <= yrs < 6
6 <= yrs < 12
12<= yrs < 18

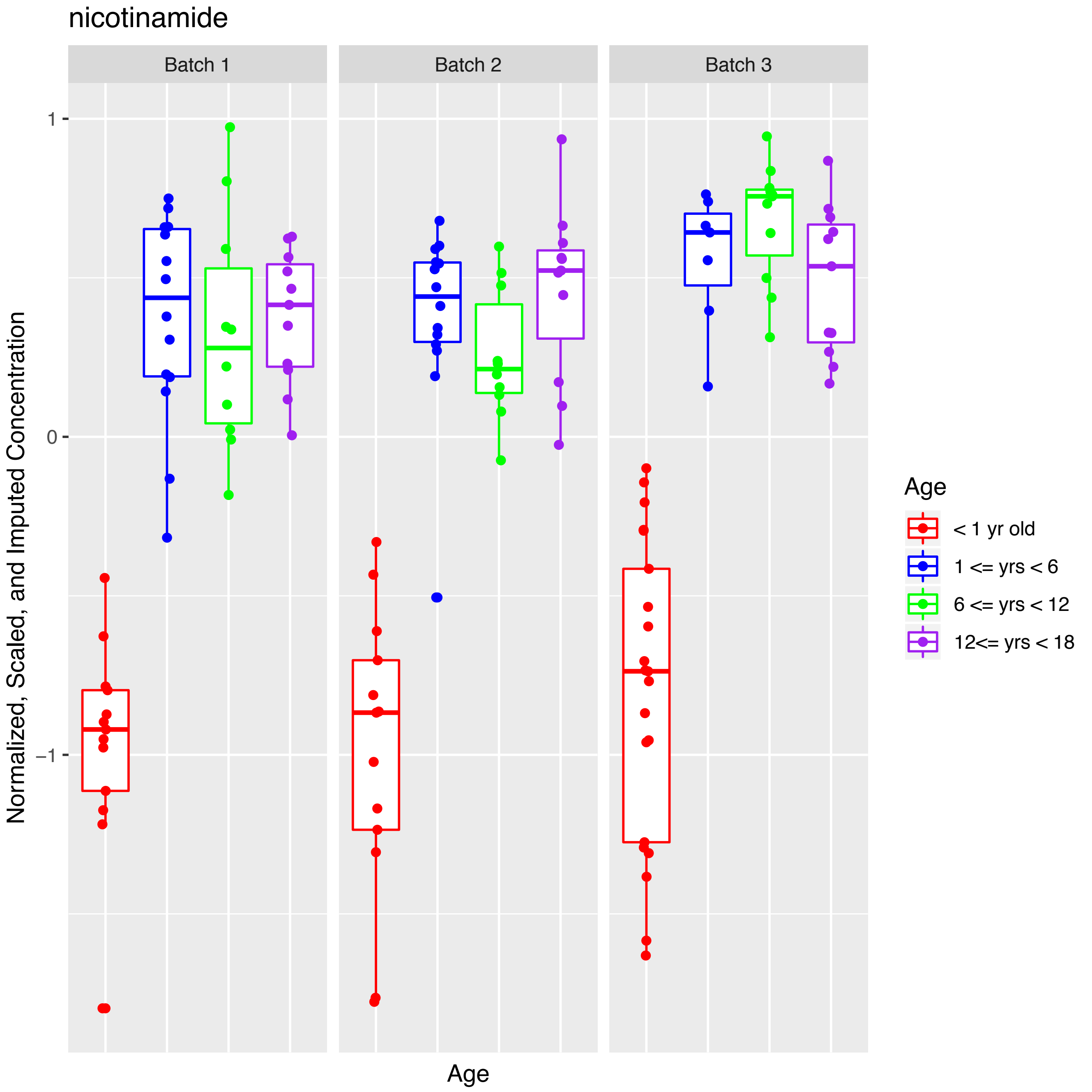
nicotinamide
Batch 1
Batch 2
Batch 3
1
0
−1
Normalized, Scaled, and Imputed Concentration
Age
Age
< 1 yr old
1 <= yrs < 6
6 <= yrs < 12
12<= yrs < 18

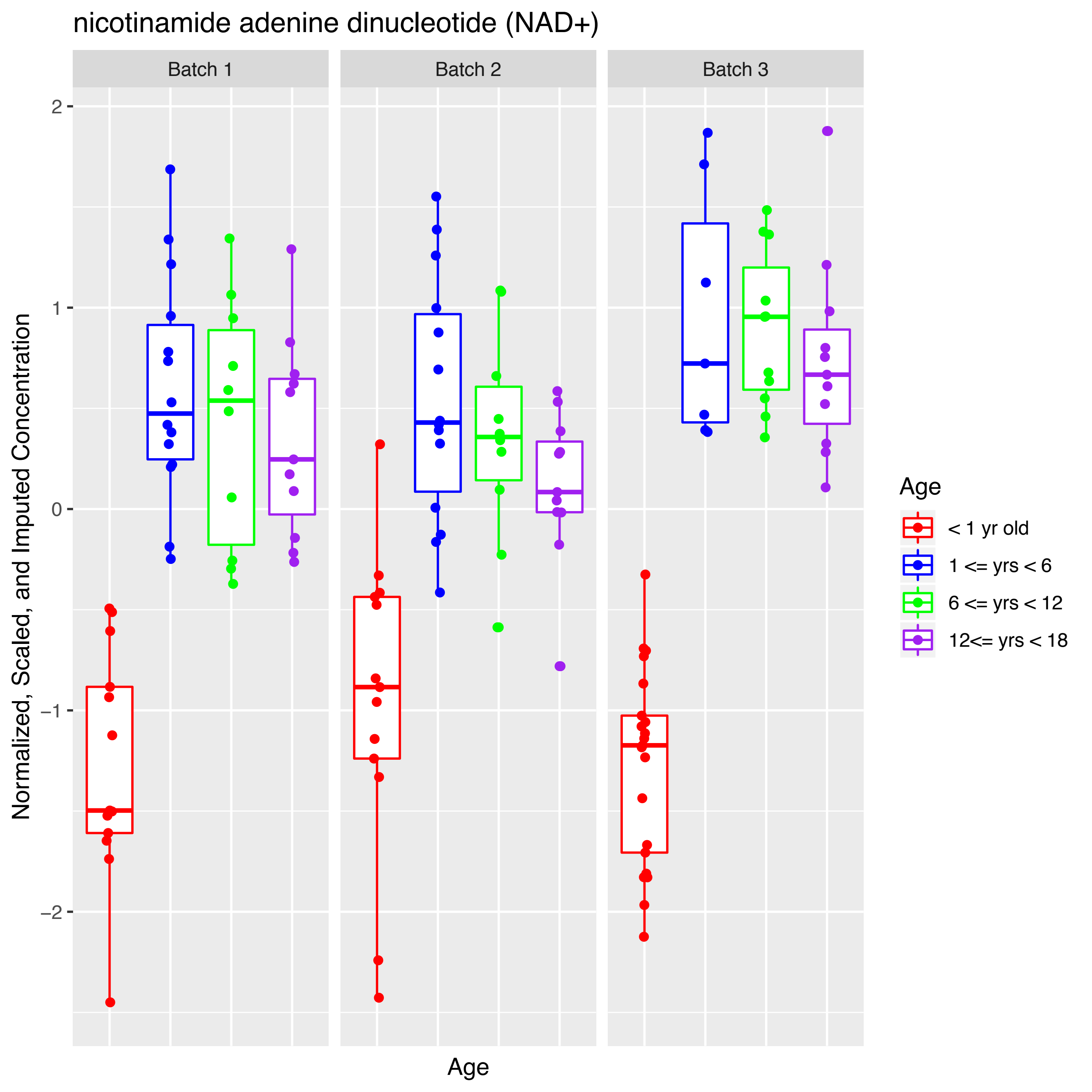

nicotinamide adenine dinucleotide (NAD+)
Batch 1
Batch 2
Batch 3
2
1
0
−1
−2
Normalized, Scaled, and Imputed Concentration
Age
Age
< 1 yr old
1 <= yrs < 6
6 <= yrs < 12
12<= yrs < 18

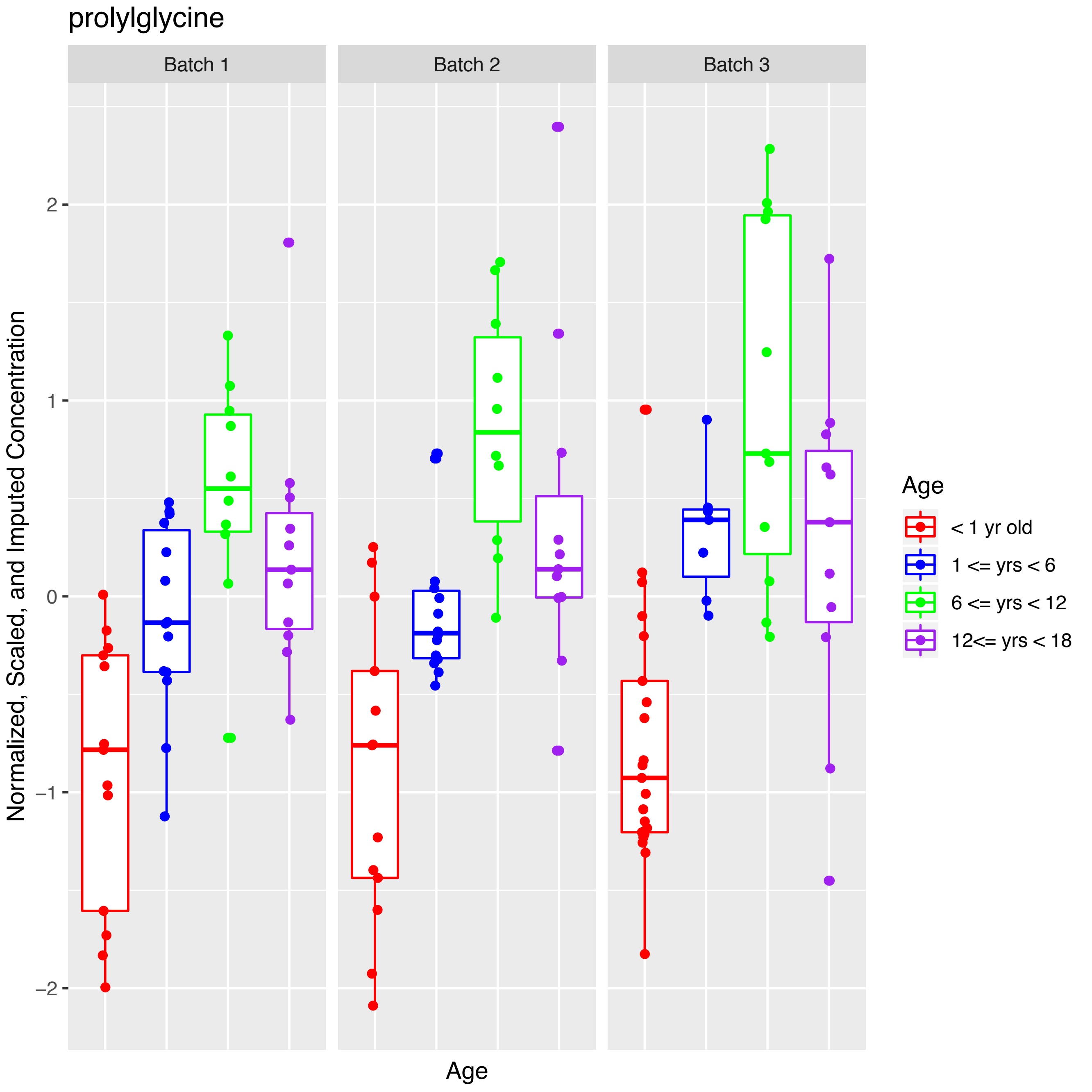
prolylglycine
Batch 1
Batch 2
Batch 3
Normalized, Scaled, and Imputed Concentration
2
1
0
−1
−2
Age
Age
< 1 yr old
1 <= yrs < 6
6 <= yrs < 12
12<= yrs < 18

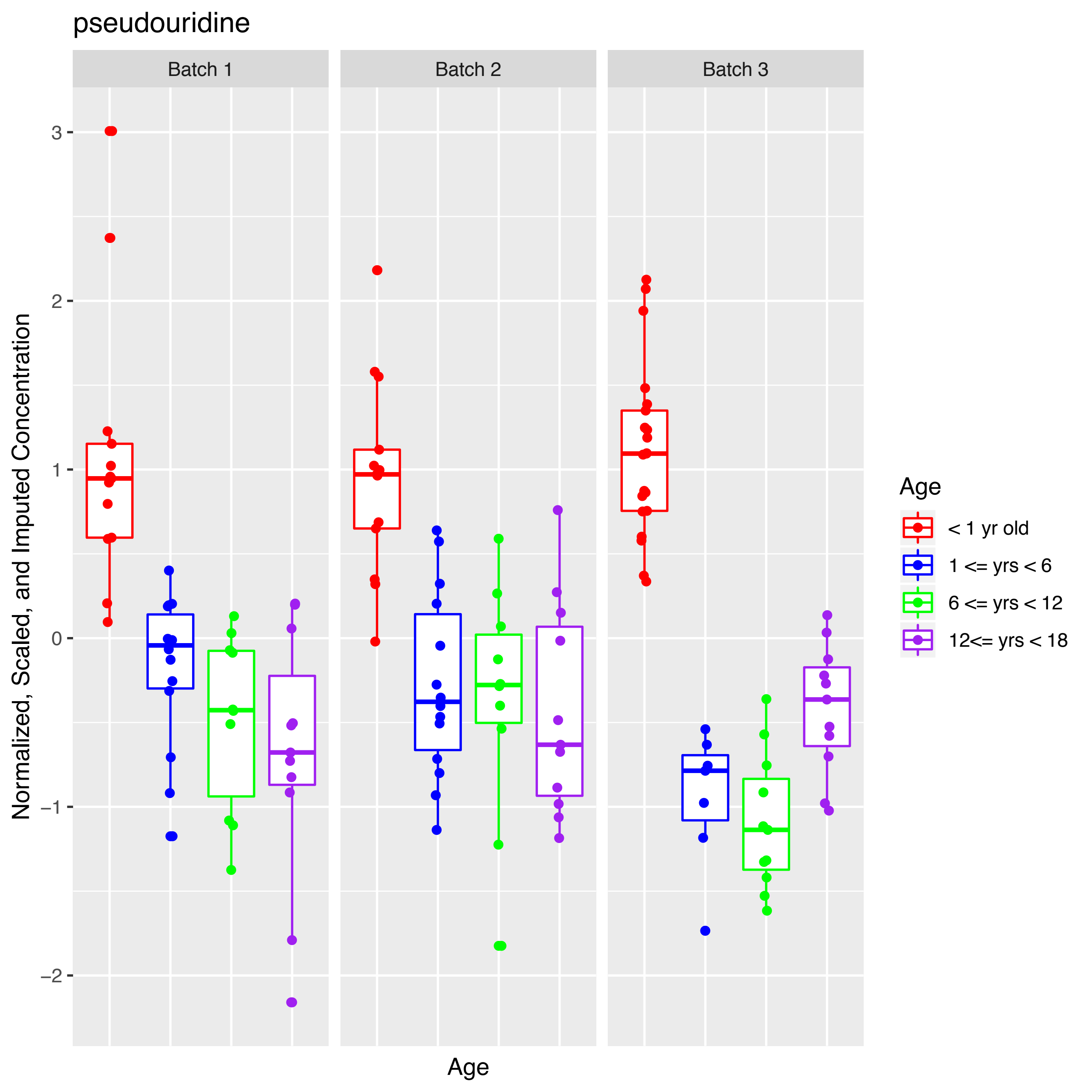

pseudouridine
Batch 1
Batch 2
Batch 3
3
2
1
0
−1
−2
Normalized, Scaled, and Imputed Concentration
Age
Age
< 1 yr old
1 <= yrs < 6
6 <= yrs < 12
12<= yrs < 18

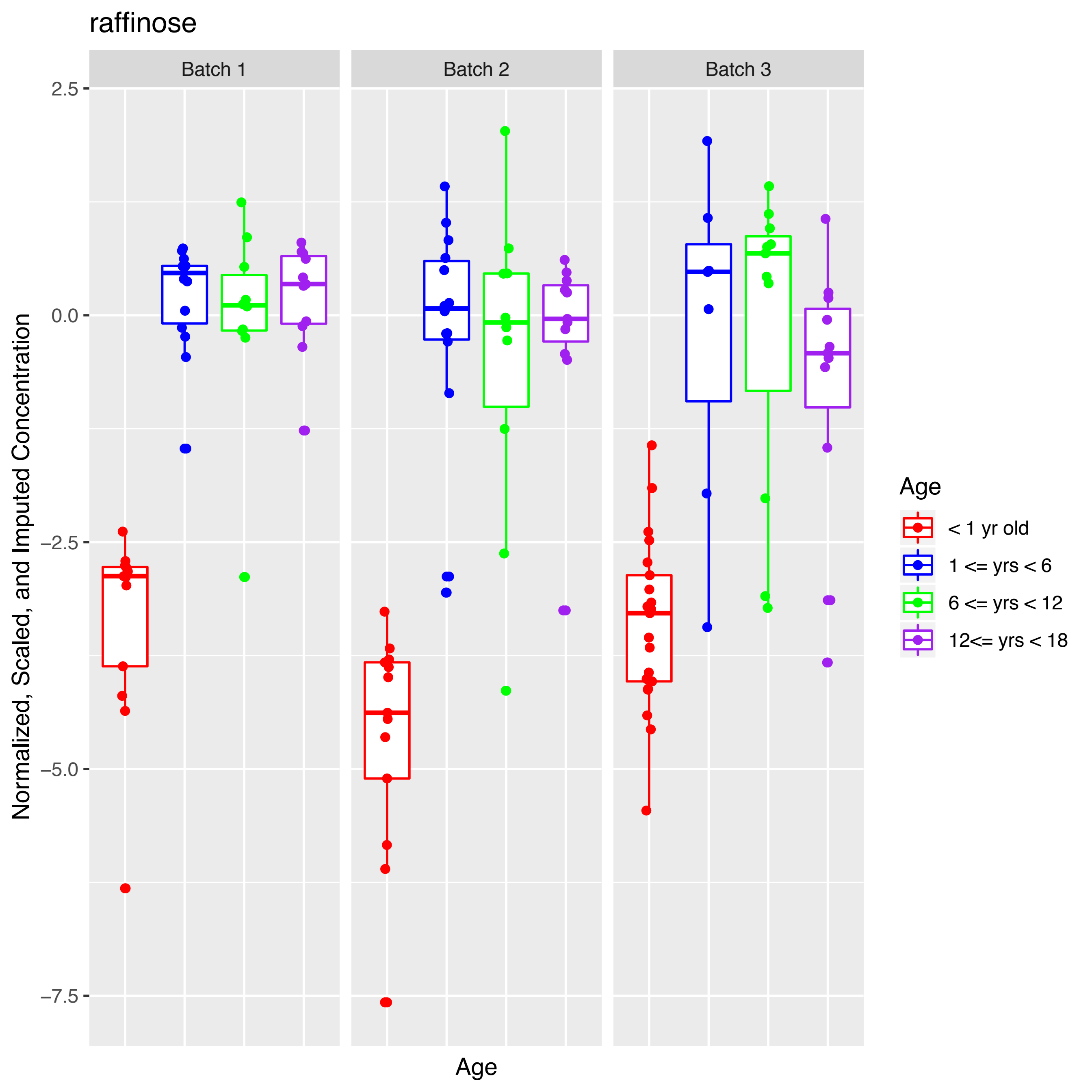

raffinose
Batch 1
Batch 2
Batch 3
2.5
0.0
−2.5
−5.0
−7.5
Normalized, Scaled, and Imputed Concentration
Age
Age
< 1 yr old
1 <= yrs < 6
6 <= yrs < 12
12<= yrs < 18

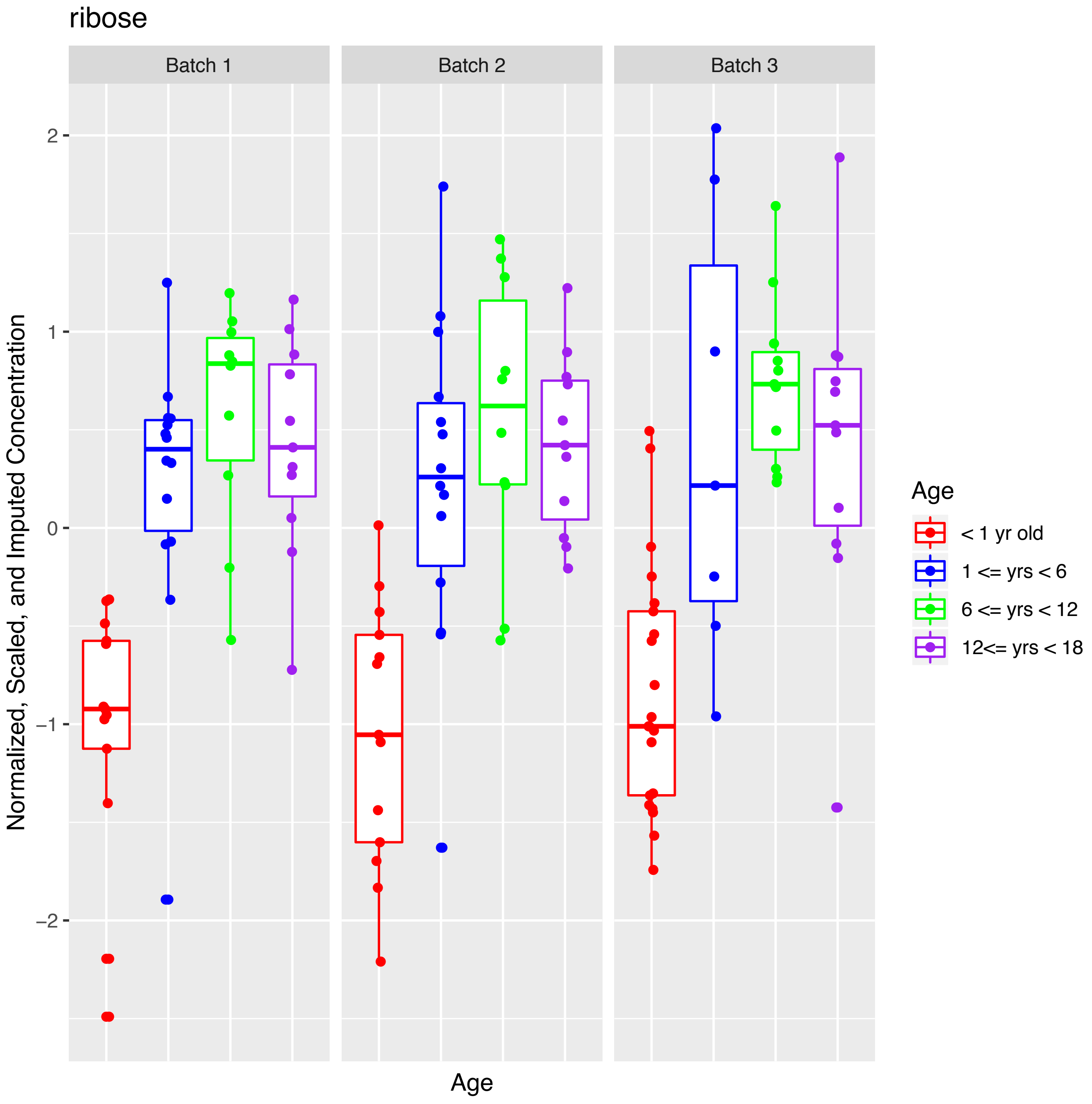

ribose
Batch 1
Batch 2
Batch 3
Normalized, Scaled, and Imputed Concentration
2
1
0
−1
−2
Age
Age
< 1 yr old
1 <= yrs < 6
6 <= yrs < 12
12<= yrs < 18

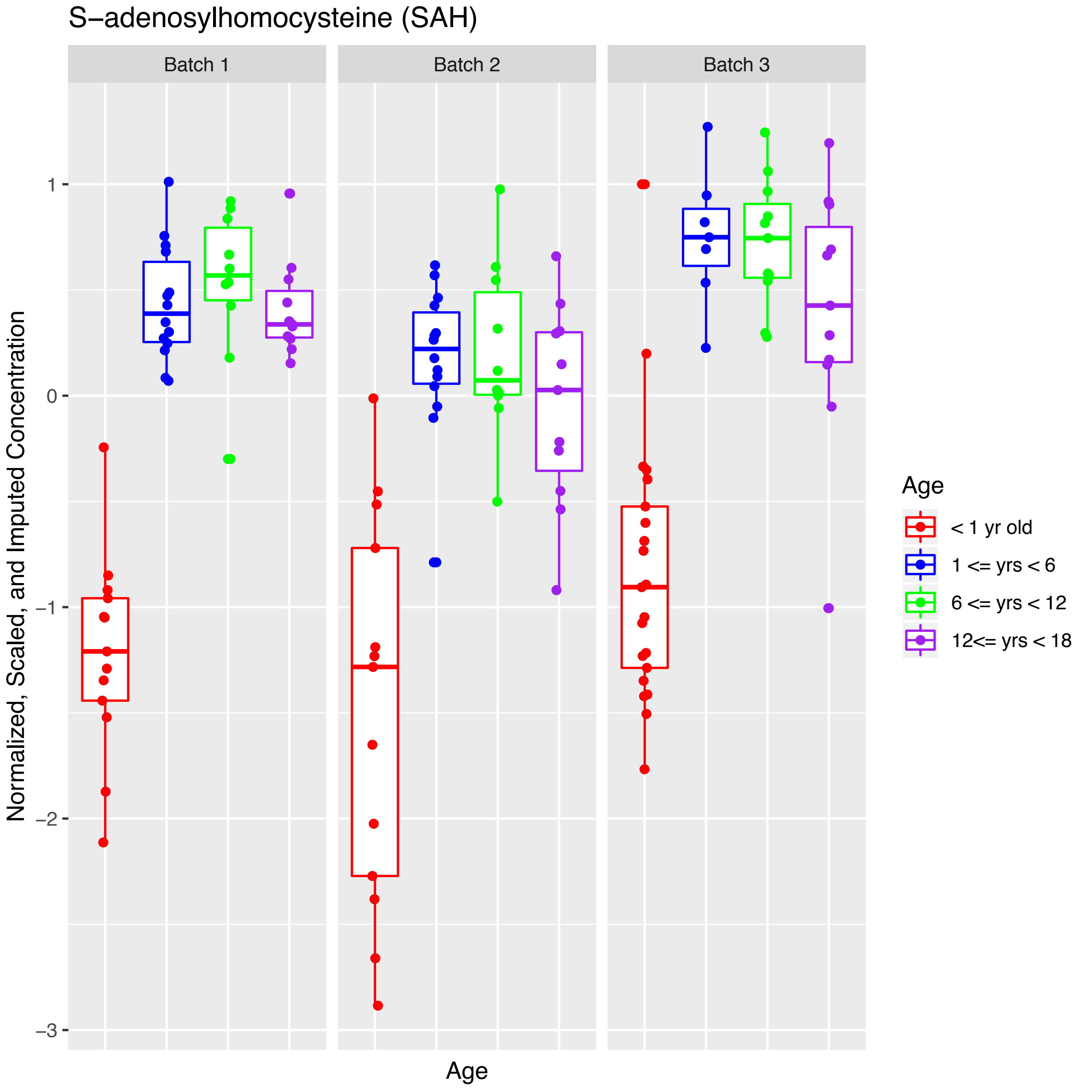

S−adenosylhomocysteine (SAH)
Batch 1
Batch 2
Batch 3
Normalized, Scaled, and Imputed Concentration
1
0
−1
−2
−3
Age
Age
< 1 yr old
1 <= yrs < 6
6 <= yrs < 12
12<= yrs < 18

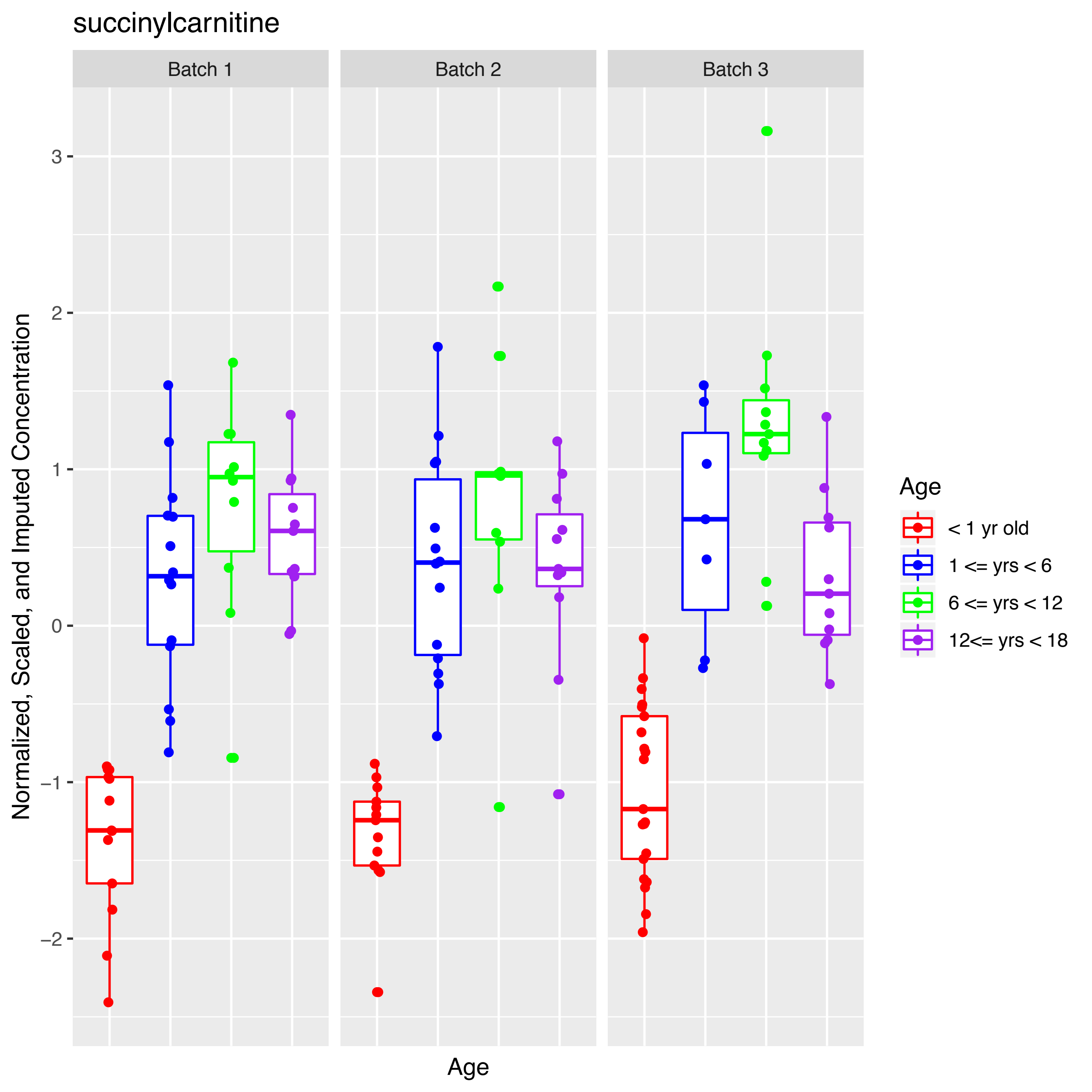
succinylcarnitine
Batch 1
Batch 2
Batch 3
3
2
1
0
−1
−2
Normalized, Scaled, and Imputed Concentration
Age
Age
< 1 yr old
1 <= yrs < 6
6 <= yrs < 12
12<= yrs < 18

taurodeoxycholate

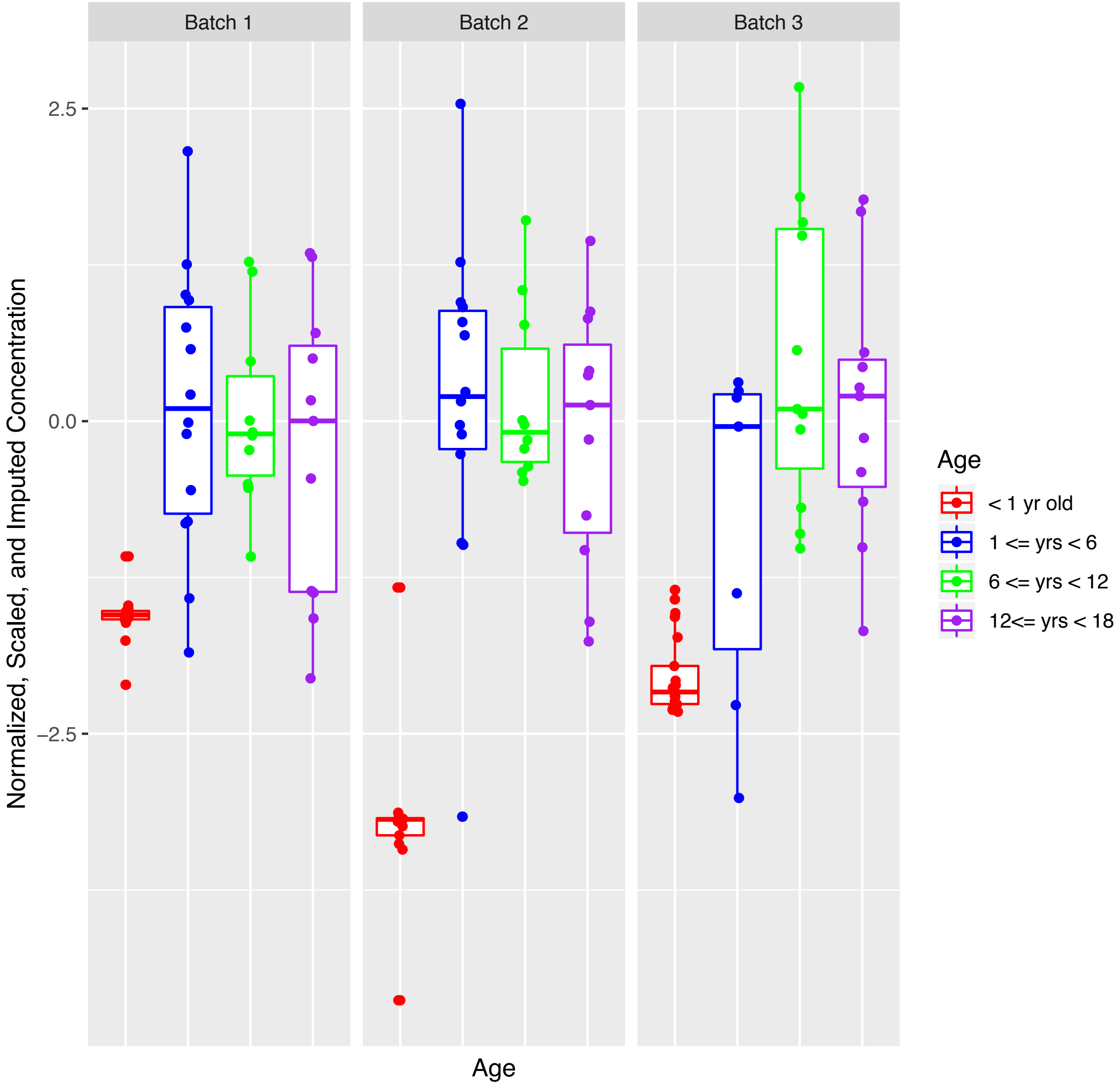

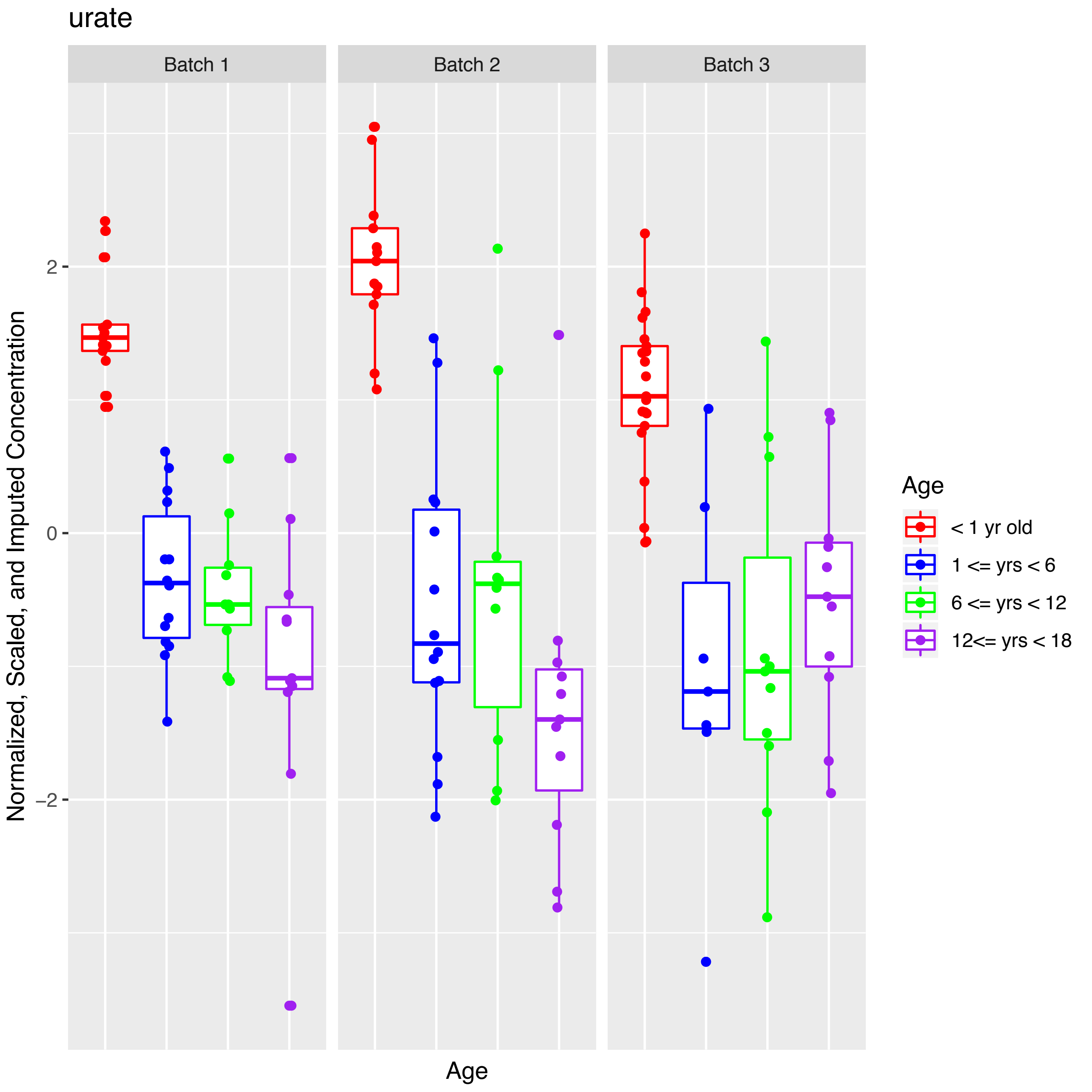
urate
Batch 1
Batch 2
Batch 3
2
0
−2
Normalized, Scaled, and Imputed Concentration
Age
Age
< 1 yr old
1 <= yrs < 6
6 <= yrs < 12
12<= yrs < 18

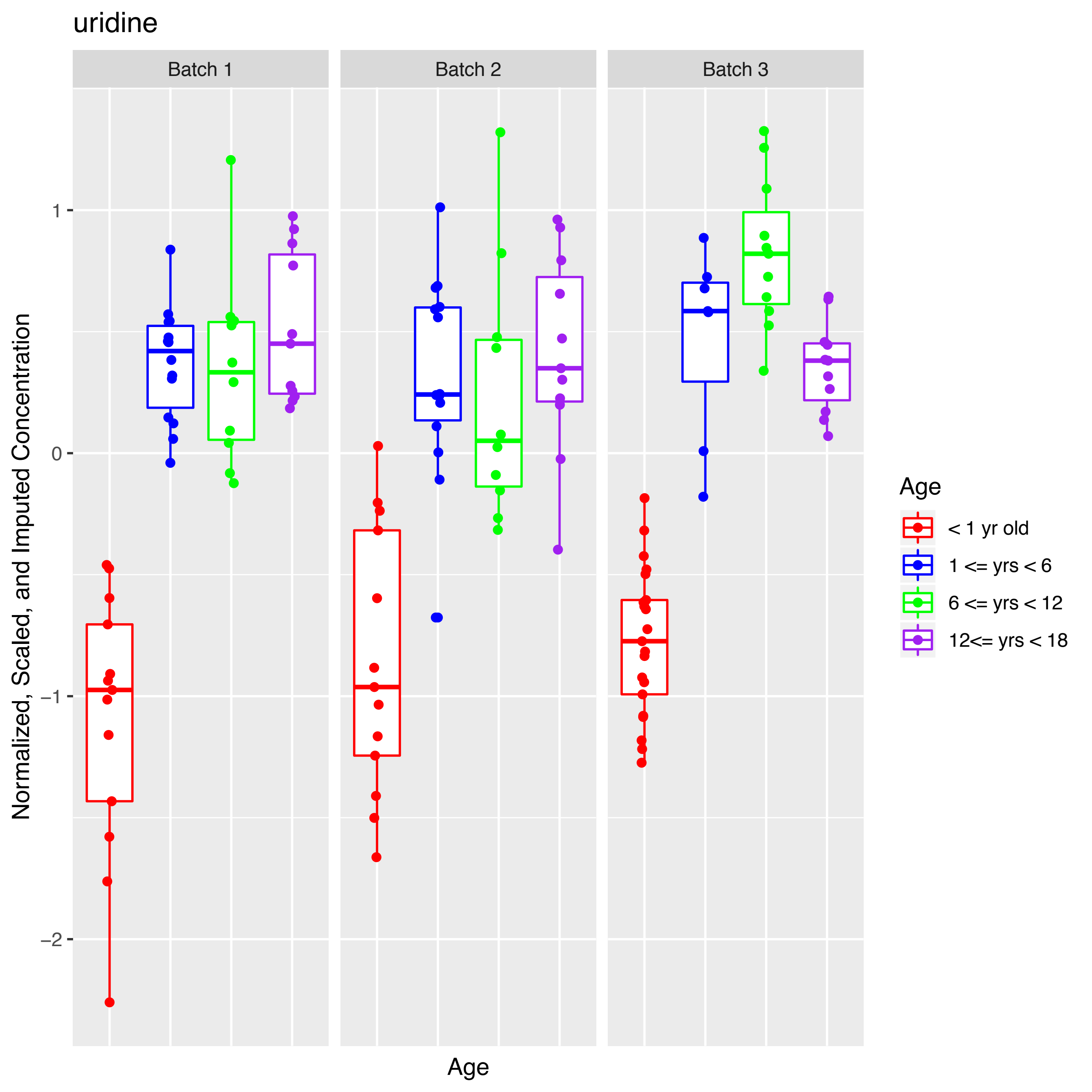

uridine
Batch 1
Batch 2
Batch 3
1
0
−1
−2
Normalized, Scaled, and Imputed Concentration
Age
Age
< 1 yr old
1 <= yrs < 6
6 <= yrs < 12
12<= yrs < 18

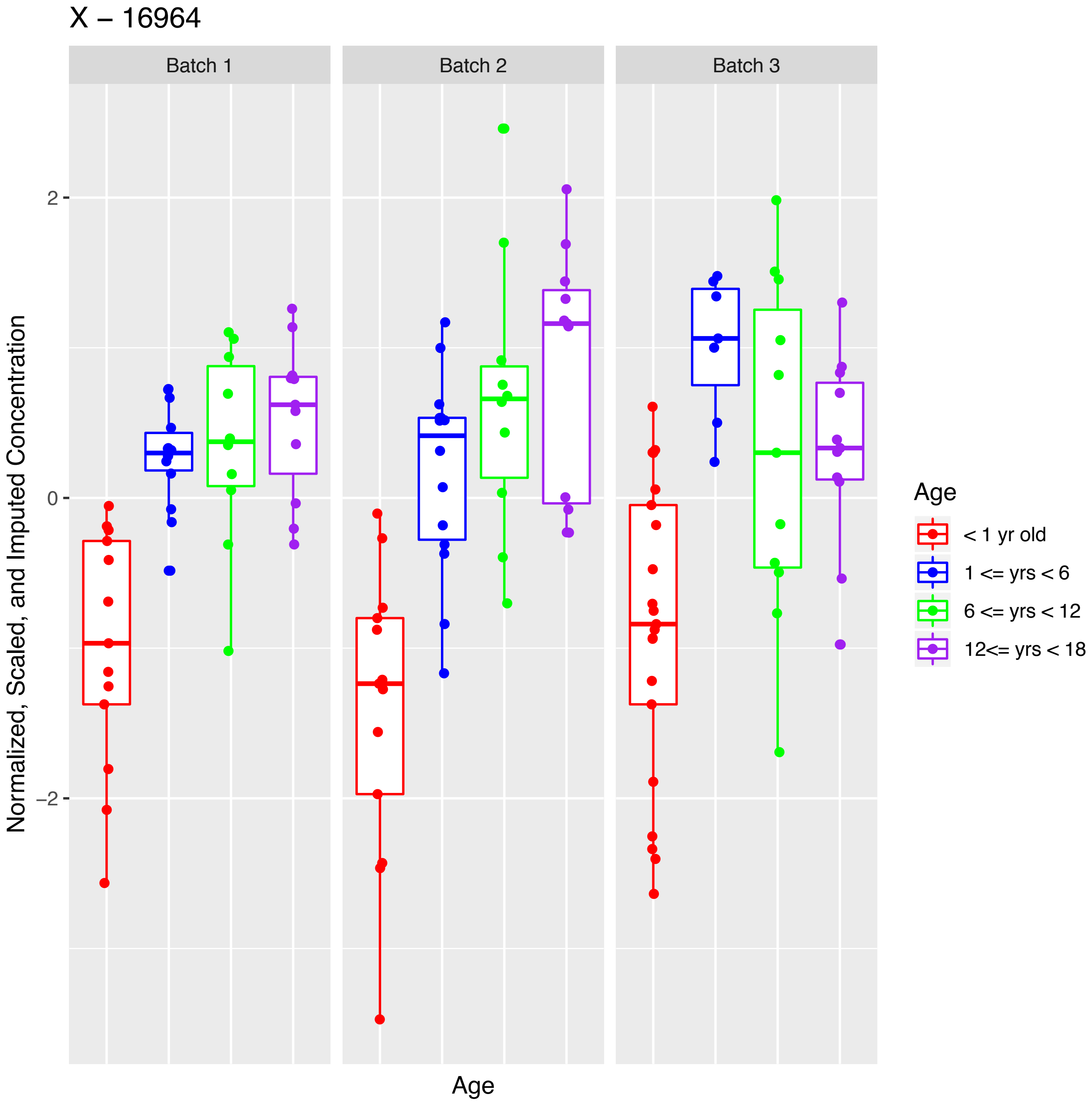
X – 16964
Batch 1
Batch 2
Batch 3
2
0
−2
Normalized, Scaled, and Imputed Concentration
Age
Age
< 1 yr old
1 <= yrs < 6
6 <= yrs < 12
12<= yrs < 18
